# New perspectives on the genetic structure of dotted gizzard shad (*Konosirus punctatus*) based on RAD-seq

**DOI:** 10.1007/s42995-024-00216-2

**Published:** 2024-02-12

**Authors:** Ying Peng, Yifan Liu, Jiasheng Li, Kun Zhang, Xun Jin, Sixu Zheng, Yunpeng Wang, Zhenming Lü, Liqin Liu, Li Gong, Bingjian Liu

**Affiliations:** 1 National Engineering Laboratory of Marine Germplasm Resources Exploration and Utilization, Zhejiang Ocean University, Zhoushan, 316022 China; 2 National Engineering Research Center for Facilitated Marine Aquaculture, Marine Science and Technology College, Zhejiang Ocean University, Zhoushan, 316022 China

**Keywords:** Population structure, *Konosirus punctatus*, SNP, RAD-seq, Local adaptation

## Abstract

To maintain, develop and rationally utilize marine organisms, understanding their genetic structure and habitat adaptation pattern is necessary. *Konosirus punctatus*, which is a commercial fish species inhabiting the Indo-west Pacific Ocean, has shown an obvious annual global capture and aquaculture production decline due to climate changes and human activities. In the present study, restriction-site associated DNA sequencing (RAD-seq) was used to describe its genome-wide single nucleotide polymorphisms panel (SNPs). Among 146 individuals collected at nine locations scattered in China, Korea and Japan, a set of 632,090 SNPs were identified. Population genetic analysis showed that *K. punctatus* individuals were divided into two significant genetic clusters. Meanwhile, potential genetic differentiation between northern and southern population of *K. punctatus* was found. Treemix results indicated that gene flow existed among sampling locations of *K. punctatus*, especially from southern Japan to others. Moreover, candidate genes associated with habitat adaptations of *K. punctatus* were identified, which are involved in diverse physiological processes of *K. punctatus* including growth and development (e.g., *KIDINS220*, *PAN3*), substance metabolism (e.g., *PGM5*) and immune response (e.g., *VAV3*, *CCT7*, *HSPA12B*). Our findings may aid in understanding the possible mechanisms for the population genetic structure and local adaptation of *K. punctatus*, which is beneficial to establish the management and conservation units of *K. punctatus*, guiding the rational use of resources, with reference significance for a profound understanding of the adaptative mechanisms of other marine organisms to the environment.

**Supplementary Information:**

The online version contains supplementary material available at 10.1007/s42995-024-00216-2.

## Introduction

Population genetic studies of marine organisms are helpful to understand their distribution, which is of special importance to managing and protecting marine population resources scientifically. Marine fish populations are rarely segregated, especially in large populations, as water mobility reduces barriers to genetic exchange between populations (Cano et al. 2008; Conover et al. 2006; Shanks et al. 2003). However, the complex geographic dynamics and hydrographic systems of the oceans have also influenced the isolation or dispersal of various marine populations, ultimately altering the genetic structure (Fernández et al. 2011; Hellberg et al. 2002). As environmental factors in marine habitats become more heterogeneous over time, the role of natural selection will shift, ultimately promoting adaptive habitat differentiation among marine populations that exhibit significant genetic differences (Nielsen et al. 2009). Moreover, climate warming and habitat destruction have now affected the genetic diversity of some small and isolated marine populations, which are more vulnerable to genetic drift and inbreeding (Ouborg et al. 2010). In short, understanding the level of genetic diversity and the mechanisms of genetic differentiation in marine organisms may contribute to the conservation and management of marine organism resources. Furthermore, it is necessary to clarify the adaptive evolution potential of different populations of marine organisms for their habitat adaptation.

*Konosirus punctatus* (Temminck and Schlegel 1846), known as the dotted gizzard shad or spotted sardine, is a suitable subject for molecular phylogeographic studies. *K. punctatus* belongs to the herring family Clupeidae (Zhang 2001). It is not only a commercial species, but is also taken as a by-catch, and is widely distributed along the coasts of China, Japan, and Korea (Liu et al. 2020). As a marine fish, *K. punctatus* can withstand freshwater conditions (Lee 1983; Myoung and Kim 2014; Song et al. 2016). The high energy conversion efficiency and decent ecological conversion efficiency make it greatly adaptable to environmental changes, ensuring the stability of the *K. punctatus* population, which plays an essential role in marine ecosystems (Yao et al. 1999). However, the annual global catch of *K. punctatus* is declining, indicating that there are some threats to its existence, particularly the degradation of estuarine ecosystems (Liu et al. 2020). Therefore, management and recovery plans are needed to prevent the collapse of *K. punctatus* populations.

With the recognition of the importance of *K. punctatus*, there has been a growing number of molecular genetic studies of its population structure*. K. punctatus* has a long pelagic larval duration (24–28 days) which may give *K. punctatus* a potential dispersal capacity and enhance the gene flow among different populations (Kong et al. 2004; Okamura and Yamada 1986). However, adult *K. punctatus* generally have a fixed migratory pattern, migrating annually only between spawning grounds along the shores of shallower waters and overwintering grounds in deeper waters, which may lead to the different genetic structures in different sea waters (Gwak et al. 2015; Liu et al. 2020; Myoung and Kim 2014; Ying 2011). For example, Myoung and Kim (2014) demonstrated that Korean populations are highly differentiated even in adjacent waters while Liu et al. (2020) found low genetic differentiation among the Chinese *K. punctatus* populations. These different structures may be caused by multiple factors, such as genetic differentiation and adaptation (Li et al. 2012). In addition, the resolution and number of markers are crucial for exploring genetic structure. For example, Ying (2011) detected the population structure and phylogeographic pattern of *K. punctatus* using four different markers [morphology, mitochondrial DNA (mtDNA) markers, amplified fragment length polymorphisms (AFLPs), and inter-simple sequence repeat (ISSR)]. These workers found morphology is less sensitive compared to the other three markers when detecting the degree of differentiation between the Japanese and Chinese populations. Meanwhile, different levels of differentiation within Chinese and Japanese populations were detected using mtDNA markers, AFLPs, and ISSR, respectively (Ying 2011). Most current markers have insufficient precision in detecting the population genetic structure of *K. punctatus* and limitations in reflecting selection or environmental influences due to quantitative limitations (Billington and Hebert 1991; Campbell et al. 2003). The advantage of restriction-site associated DNA sequencing (RAD-seq) technology is that it can efficiently detect genome-wide single-nucleotide polymorphisms (SNPs) even at low sequencing depth, which ultimately provides an efficient and accurate solution for studying the genetic structure of a species, even those with low genetic differentiation (Zhang et al. 2019). Simple library construction, short duration of the experiment, and well-developed data processing and analysis pipelines have made the RAD-seq technology popular in SNP identification and population genetics studies (Eaton and Ree 2013; McCormack et al. 2013). Furthermore, RAD-seq technology has been successfully applied to detect SNPs in the whole genome of numerous marine species, such as *Mugil cephalus* (Krück et al. 2013), *Carcinus maenas* (Jeffery et al. 2017) and *Setipinna tenuifilis* (Peng et al. 2021), to provide more reliable evidence for studying their genetic structure and adaptive evolution (Miller et al. 2007). However, no population genetics study has been conducted to detect genome-wide SNPs of *K. punctatus* based on RAD-seq. Therefore, a comprehensive population genetic analysis of *K. punctatus* based on SNPs via RAD-seq is needed.

In the present study, individuals of *K*. *punctatus* were collected from nine sampling locations along the coasts of China, Japan, and Korea. RAD-seq was first used to identify genome-wide SNPs in *K. punctatus.* Population genomic methods have been used to identify genetic variation and explore the adaptive evolution of *K. punctatus* populations. Finally, the results of this study will improve our understanding of the population structure and habitat-adaptive differentiation of *K. punctatus*.

## Materials and methods

### Collection and RAD-seq of *K. punctatus* samples

**Fig. 1:**
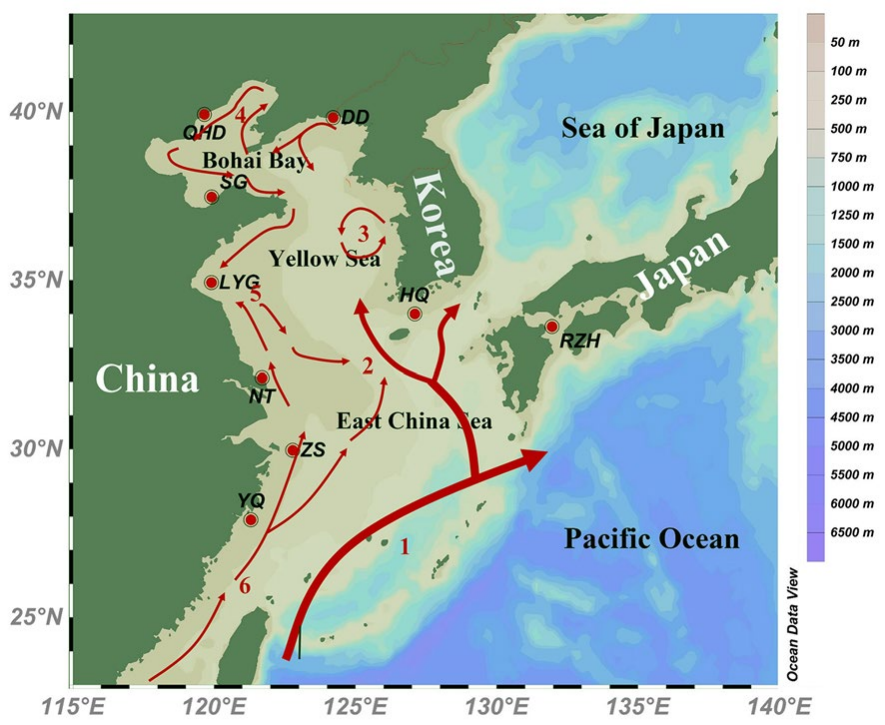
Map of the studied area depicting sampled populations and currents. 1 Kuroshio Current, 2 Yellow Sea Warm Current, 3 Yellow Sea Cold Water Mass, 4 Bohai Circulation, 5 China Coastal Current, 6 South China Sea Warm Current. Chinese populations: Qinhuangdao (QHD), Dandong (DD), Shouguang (SG), Lianyungang (LYG), Nantong (NT), Zhoushan (ZS), and Wenzhou (YQ). Korean population: Jeolla-Do (HQ). Japanese population: Saga (RZH)

We collected samples of 146 *K. punctatus* from nine locations, including Qinhuangdao (QHD), Dandong (DD), Shouguang (SG), Lianyungang (LYG), Jeolla-Do (HQ), Saga (RZH), Nantong (NT), Zhoushan (ZS), and Wenzhou (YQ) along the coasts of China, Japan, and Korea (Fig. 1; Supplementary Table S1). Muscle tissue from each fish was preserved in 95% ethanol for DNA extraction. Total genomic DNA was extracted using a standard procedure (Dhaliwal 2013). DNA completeness and concentration were checked by gel electrophoresis and spectrophotometry. The standard for high-quality extracted DNA was that the range of OD_260/280_ was between 1.6 and 1.8. At the same time, the electrophoretic bands are well-defined and there is no significant dispersion.

After that, high-quality genomic DNA was used to construct the RAD library (Etter et al. 2011). Firstly, enzyme digestion was performed after the restriction endonuclease was added to each genomic DNA sample. P1 adapters were added to the enzymatically digested fragments. Enzymatically digested fragments ligated to the P1 adapter were mixed and end-repaired. Then, dA and Illumina P2 adapters were added to the mixture fragments. PCR was employed to amplify the fragments with P1 and P2 adapters. Finally, paired-end sequencing was conducted on the reads of the library (150 bp) through the Illumina Novaseq 6000 sequencing platform.

### SNP calling

Raw data was acquired and subjected to a quality control step. The Cutadapt program (version 2.6) (Martin 2011) was used to remove low-quality read pairs following the parameter listed below: -q 20 (trim low-quality reads), -e 0.1 (maximum allowed error rate by keeping the default value 0.1 = 10%), -n 1 (remove up to one adapter from each read) and -m 20 (discard trimmed reads that are shorter than 20 bp). Then the clean reads were aligned with the reference genome of *K. punctatus* at NCBI (Genome accession number: PRJNA664710, scaffold N50: 32.23 Mb, BUSCO completeness: 93.54%) (Liu et al. 2022a) using the algorithm of BWA-MEM in the BWA software (Li and Durbin 2010) with the default settings. After that, SAMtools (Li et al. 2009) was used to sort reads. Duplicates formed in PCR were cleared using the Picard tool (Zhao 2018) in the Java program. GATK (McKenna et al. 2010) was applied for SNP calling. SNPs were selected using VariantFiltration according to the following conditions: QD < 2.0, MQ < 40.0, FS > 60.0, SOR > 3.0, MQRankSum < − 12.5, ReadPosRankSum < − 8.0. Finally, VCFtools (Danecek et al. 2011) was performed to filter SNPs with the parameters: -maf 0.05; -max-missing 0.9; -minQ 30; -min-alleles 2 and -max-alleles 2. The filtered SNPs were used for subsequent analysis.

### Population genetic analysis

The genetic parameters of nine sampling locations were statistically performed for *K. punctatus*, including polymorphic loci, the nucleotide diversity (*π*), observed heterozygosity (*H*_O_), expected heterozygosity (*H*_E_), the inbreeding coefficient (*F*_IS_), and pairwise* F*_ST_ values (Meirmans and Hedrick 2011) by the “populations” module of Stacks software (Catchen et al. 2013).

### Population stratification and genetic structure

Linkage disequilibrium (LD) filter was performed using VCFtools (Danecek et al. 2011) before population structure analysis according to the criteria: -indep-pairwise 50 10 0.2. Briefly, principal component analysis (PCA) analysis was performed through the Plink program (Purcell et al. 2007) based on 259,449 SNPs. The contribution ratios for each principal component were calculated and the PCA diagram was drawn in the R software (Team RC 2013) environment. Furthermore, the admixture history and the cross-validation error for the hypothetical runs from *K* (the number of ancestral populations) = 1–4 were determined using the Admixture program (Alexander et al. 2009). The optimal *K*-value was determined through the cross-validation (CV) error plot using the received log data. Finally, R software (Team RC 2013) was used to draw admixture plots using the *Q* estimate files created by the Admixture programs. In addition, the phylogenetic tree was constructed based on the Neighbor-joining (NJ) method. First, a Perl script was used to convert the VCF file into a PHYLIP file (Retief 2000). A configuration file was then prepared for the dnadist program in the PHYLIP package to run the distance matrix. Finally, the NJ tree was formed through the Neighbor program in PHYLIP (Saitou and Nei 1987). The image of the phylogenetic tree was beautified using iTOL (Letunic and Bork 2021). To precisely resolve the genetic relationships among 146 *K. punctatus* individuals, the R package NETVIEW (Steinig et al. 2016) was used to construct a genetic relationship network map for them. Similarly, the number of mutual nearest neighbor graphs (mkNNGs) needs to be determined before running NETVIEW. It is essential to find a network topology within the possible range of mkNNGs that presents the genetic similarity among all individuals at an appropriate resolution.

### Gene flow and population history

To find the genetic drift and infer gene flows of historical population splits and mixtures among the nine sampling locations of *K. punctatus*, Treemix software (Pickrell et al. 2012) was applied using Fitak (2021)’s methods. Population history was modeled from zero to ten migration events (1 ≤ *m* ≤ 10) and run for 10 replicates with the RZH as the root. To avoid converging on the same composite likelihood for each replicate, the number of SNPs per window was varied across runs from 100 to 1000 in 50 SNP increments. Once completed, the Treemix output files were analyzed with OptM v0.1.5 (Fitak 2021) using default parameters. Finally, the tree was visualized using the R script embedded in Treemix.

In addition to the above method of detecting population history dynamics by inferring gene flow, we also inferred the dynamic history of population contraction or expansion of *K. punctatus* based on the Site Frequency Spectrum (SFS). First, the SFS was calculated by the ANGSD 0.935 software (Korneliussen et al. 2014) based on the bam file and filtered with the parameter "-minMapQ 30 -minQ 20" for low-quality maps. Then, the likelihood of SFS was evaluated using -doSaf based on individual genotype likelihoods to obtain the.Saf file. Next, the realSFS was employed to generate the folded SFS based on the estimation of maximum likelihood. Finally, Stairway plot 2 (Liu and Fu 2020) was applied to infer the effective population size (*N*e) of *K. punctatus* populations under a mutation rate of 1.29e^−9^ per generation and a generation time of one and a half years.

### Detection of loci under putative selection and gene annotation

To clarify the mechanisms of local adaptation for *K. punctatus* populations, we combined the redundancy analysis (RDA) (Forester et al. 2018) and BayeScan methods (Foll et al. 2008). RDA as a genotype-environment association method was applied to evaluate the percentage of genomic variation interpreted by environmental variables and to detect SNPs subject to environmental selection (Lasky et al. 2012; Waits and Sork 2010). The principle of the method is to perform multiple linear regression analysis (MLR) on genetic and environmental data through the RDA function of the R package vegan 2.6 and generate a matrix of fitted values (Oksanen et al. 2007; Zulliger et al. 2013). Then PCA of the fitted values was performed to produce canonical axes, which were linear combinations of the factors (Legendre and Legendre 2012). The main steps were as follows: first, the allele frequencies of 259,449 SNPs were assessed through Plink v 1.9 (Purcell et al. 2007) software and the missing values in the plink. The raw file was replaced with the most common genotypic substitution at the same position in all individuals. Second, 19 marine environmental variables were collected through the Bio-ORACLE database (Assis et al. 2018), including mean seawater temperature (*T*_avg_), mean warmest seawater temperature (*T*_max_), mean coldest seawater temperature (*T*_min_), mean seawater salinity (*S*_arg_), mean highest seawater salinity (*S*_max_), mean lowest seawater salinity (*S*_min_), mean dissolved oxygen (*O*_arg_), mean maximum dissolved oxygen (*O*_max_), mean minimum dissolved oxygen (*O*_min_), mean pH (pH_arg_), mean water flow rate (*C*_arg_), mean fastest water flow rate (*C*_max_), mean slowest water flow rate (*C*_min_), mean nitrate content (*N*_arg_), mean maximum nitrate content (*N*_max_), mean minimum nitrate content (*N*_min_), mean phosphate content (Pho_arg_), mean maximum phosphate content (Pho_max_), and mean minimum phosphate content (Pho_min_). Third, the ArcGIS software (Wampler et al. 2013) was then used to extract the marine environmental data of the *K. punctatus* sampling sites. Due to the correlation between 19 environmental variables, one of the two highly related (|*r*| > 0.7) environmental variables was removed using Pearson correlation analysis to avoid environmental redundancy in RDA. Five environmental variables were retained, including *T*_max_, pH_avg_, *C*_max_, *N*_max_, and Pho_max_ (Supplementary Fig. S1; Supplementary Table S2). The significances of global RDA and constrained axes were assessed using 1000 permutations and a *P*-value threshold of 0.05. Finally, we screened out outlier SNPs loading ± 3 SD from the mean loading of significant RDA axes and classified SNPs associated with different environmental variables (Forester et al. 2018). In addition to RDA, a Bayesian method implemented in BayeScan version 2.1 (Foll et al. 2008) was used to identify outliers. BayeScan was run for 20 pilot runs with a length of 5000 iterations after a burn-in of 50,000 steps with prior odds of 1000. SNPs with a false discovery rate (FDR) lower than 0.01 were considered outliers.

To more precisely identify loci associated with the adaptive evolution of *K. punctatus* populations, we intersected the loci screened by BayeScan 2.1 and RDA analysis to statistically identify highly differentiated SNPs that were significantly related to different environmental variables. Then, the BEDTools (Quinlan and Hall 2010) was applied to extract genes in the 10 kb upstream and downstream range of these loci, and eggNOG-mapper (Cantalapiedra et al. 2021) was employed to annotate these genes. Finally, the screened environmentally associated genes were enriched for Gene ontology (GO) and Kyoto Encyclopedia of Genes and Genomes (KEGG) functions using ClusterProfiler (Yu et al. 2012). Only the top 25% of pathways with *P* < 0.05 were retained when GO enrichment was performed, and only the top 50% of pathways were retained when KEGG enrichment was conducted. In addition, we screened genes with multiple environmental associations from the above genes for subsequent discussion.

## Results

### RAD-seq and SNP identification

We collected 146 *K. punctatus* individuals from nine locations along the coasts of China, Japan, and Korea (Fig. 1; Supplementary Table S1). RAD-seq of 146 *K. punctatus* individuals generated 1468 million paired-end data, ranging from 6,431,148 to 15,838,216 (Supplementary Table S3). After the low-quality filtering step, 1,466,640,306 reads were retained (99.9%). Generally, the GC content of filtered clean reads was between 41.81% and 42.06%, which was relatively stable (Supplementary Table S3). Quality scores ranged from 97.68% to 97.99% for Q20, and 93.15% to 94.30% for Q30, indicating the high quality of RAD sequencing results. Most mapping rates of clean reads were higher than 97% (Supplementary Table S3). After individual sequence alignment and SNP extraction, a total of 6,510,125 pre-filtered SNPs were eventually detected. When the low-quality SNPs were excluded, a total of 632,090 SNPs were obtained over the whole genome of the *K. punctatus* populations. After linkage disequilibrium (LD) filtering, 259,449 SNPs were obtained for population genetic structure analysis.

### Genomic diversity of *K. punctatus*

We calculated the diversity index of nine locations for *K. punctatus* based on 632,090 SNPs (Table 1). The whole-genome level of genetic diversity of nine *K. punctatus* locations was evaluated. The results indicated values of *H*_E_ (0.17–0.25), *H*_O_ (0.17–0.25), *F*_IS_ (0.03–0.10), and *π* (0.18–0.26) of the nine locations, which showed the lowest value in the RZH (Table 1). Furthermore, percentages of polymorphic loci (80.35–95.60%) for other *K. punctatus* sampling locations were high except for RZH (59.53%) (Table 1).

**Table 1:** Statistics of diversity parameters

Pop ID	*H*_O_	*H*_E_	*π*	*F*_IS_	Polymorphic_Loci (%)
QHD	0.22	0.24	0.25	0.10	89.06
DD	0.24	0.25	0.26	0.07	92.72
SG	0.24	0.24	0.26	0.04	83.45
LYG	0.25	0.24	0.26	0.03	80.35
NT	0.23	0.25	0.25	0.07	93.99
ZS	0.22	0.24	0.24	0.07	91.35
YQ	0.23	0.25	0.26	0.08	93.12
HQ	0.24	0.25	0.26	0.07	95.60
RZH	0.17	0.17	0.18	0.03	59.53
Mean	0.23	0.24	0.25	0.06	86.57

### Genetic differentiation levels and genetic structure of *K. punctatus*

The fixation index (*F*_ST_) evaluation was performed to assess levels of genetic differentiation among nine sampling locations for *K. punctatus* (Meirmans and Hedrick 2011). In practice, low differentiation is indicated when *F*_ST_ ranges from 0.00 to 0.05 while 0.05–0.15 indicates moderate differentiation, and a high level of differentiation exists when *F*_ST_ > 0.15 (Govindaraju 1989). The *F*_ST_ among nine *K. punctatus* sampling locations ranged from 0.01 (between YQ and NT) to 0.17 (between ZS and RZH). Generally, pairwise *F*_ST_ values were low among different locations, especially between YQ and NT, indicating free interbreeding between them (Table 2). It should be noted that *F*_ST_ values between the RZH and other locations were all larger than 0.13, indicating a large genetic distance between them (Table 2).

**Table 2:** Estimated pairwise *F*_ST_ value in stacks

	DD	SG	LYG	NT	ZS	YQ	HQ	RZH
QHD	0.02	0.03	0.03	0.02	0.03	0.02	0.02	0.15
DD		0.02	0.02	0.02	0.03	0.02	0.02	0.14
SG			0.03	0.02	0.04	0.02	0.02	0.15
LYG				0.02	0.03	0.02	0.02	0.16
NT					0.02	0.01	0.02	0.15
ZS						0.02	0.03	0.17
YQ							0.02	0.15
HQ								0.13

**Fig. 2:**
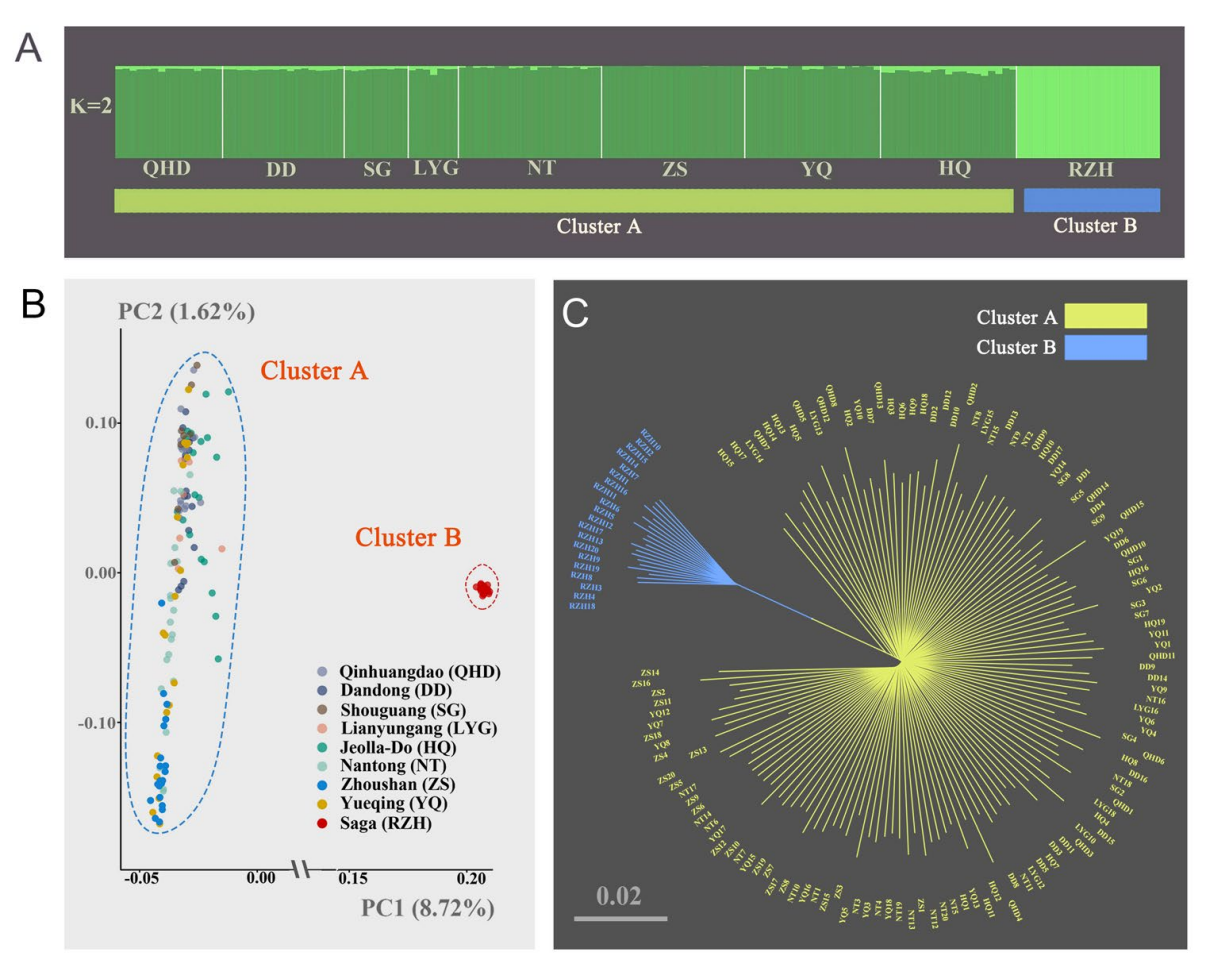
Population structure and genetic differentiation based on 259,449 SNPs. **A** Population structure. The length of each colored segment represents the proportion of the individual genome inferred from ancestral populations (*K* = 2). **B** Principal component analysis. **C** NJ tree showing the phylogenetic relationships. Chinese populations: Qinhuangdao (QHD), Dandong (DD), Shouguang (SG), Lianyungang (LYG), Nantong (NT), Zhoushan (ZS), and Wenzhou (YQ). Korean population: Jeolla-Do (HQ). Japanese population: Saga (RZH)

Furthermore, the analysis of population structure among different locations via the Admixture program (Alexander et al. 2009) revealed that the best *K*-value was 2, which indicated that 146 *K. punctatus* individuals used in this study belonged to two clusters (Cluster A and Cluster B) (Supplementary Fig. S2). Individuals in Cluster B were highly differentiated from other individuals in Cluster A (Fig. 2A). The principal component analysis (PCA) results indicated the presence of two subgroups among 146 accessions in our study (Fig. 2B), which was consistent with the *K* = 2 analysis of the population structure analysis (Fig. 2A). The first and second coordinates explained 8.72% and 1.62% of the variation, respectively. The PCA plot showed that all samples were divided into two populations (Cluster A and Cluster B) along the first discriminant function (PC1) while individuals from Cluster A were evenly distributed along the second discriminant function (PC2) (Fig. 2B). What is more, the Neighbor-joining (NJ) tree also showed that Cluster B was highly differentiated from Cluster A (Fig. 2C), and individuals from Cluster A were mixed and lowly differentiated, which was consistent with the Admixture and PCA cluster results (Fig. 2A, B).

**Fig. 3:**
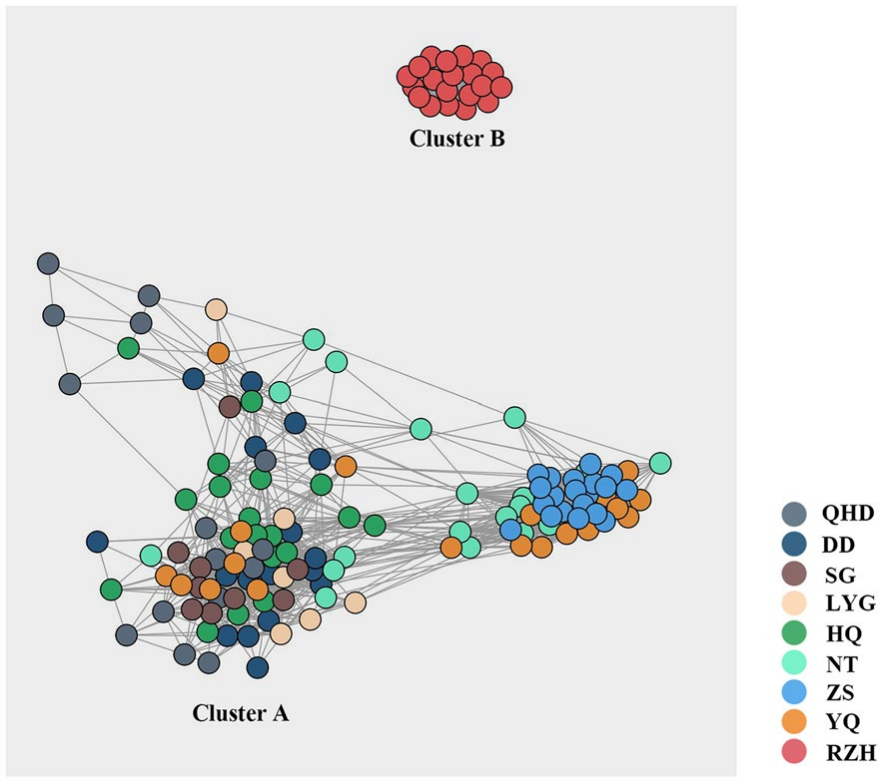
Network of *K. punctatus* based on 259,449 SNPs, color shades represent sampling locations (*K* = 35, *n* = 146). Chinese populations: Qinhuangdao (QHD), Dandong (DD), Shouguang (SG), Lianyungang (LYG), Nantong (NT), Zhoushan (ZS), and Wenzhou (YQ). Korean population: Jeolla-Do (HQ). Japanese population: Saga (RZH)

To achieve a finer resolution of genetic relationships among 146 *K. punctatus*, multiple algorithms of NETVIEW (Fast-Greedy, Infomap, Walktrap) were applied to pick the best *K* value in the range of *K* = 1 to 100 (Steinig et al. 2016). As shown in Supplementary Fig. S3, the results of all algorithms were generally consistent, which showed that the decline was flat at *K* = 35 and the mutual nearest neighbor graphs (mkNNGs) gradually emerged as a fine genetic structure (Fig. 3). Therefore, the genetic network structure among *K. punctatus* individuals was constructed at *K* = 35. A high degree of differentiation was also detected between Cluster A and Cluster B (Fig. 3), which agreed with plots of Admixture, PCA and NJ tree (Fig. 2). In addition, details of the clustering among individuals of Cluster A showed that most individuals sampled from the QHD, DD, SG, LYG, and HQ locations clustered on the left side of the genetic network graph while most individuals sampled from the NT, ZS and YQ locations clustered on the right side of the genetic network graph (Fig. 3). The results suggested that although the kinship of individuals in Cluster A was close, there was a potential differentiation in Cluster A and there may be a hidden genetic structure among these individuals.

### Gene flow among *K. punctatus* sampling locations

**Fig. 4:**
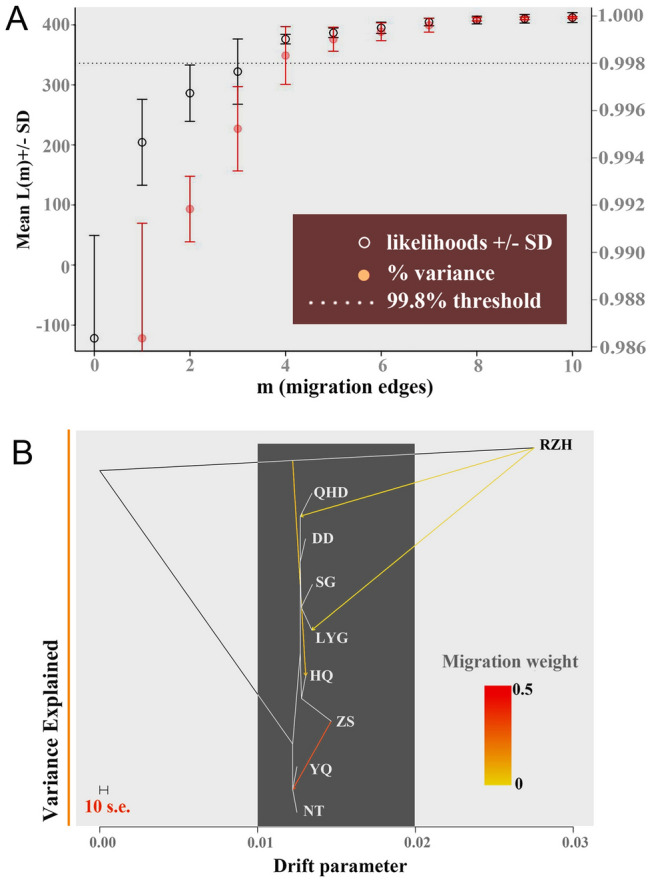
Gene flow and Spread history of *K. punctatus*. **A** The output produced by OptM for the simulated dataset with *m* = 4 migration edges. The mean and standard deviation (SD) across 10 iterations for the composite likelihood *L*(*m*) (left axis, black circles) and proportion of variance explained (right axis, red “*x*” s). The 99.8% threshold (horizontal dotted line) is that recommended by Pickrell and Pritchard. **B** A population-based maximum likelihood Treemix analysis. Branch lengths are equivalent to a genetic drift parameter. Chinese populations: Qinhuangdao (QHD), Dandong (DD), Shouguang (SG), Lianyungang (LYG), Nantong (NT), Zhoushan (ZS), and Wenzhou (YQ). Korean population: Jeolla-Do (HQ). Japanese population: Saga (RZH)

We inferred the history of gene flow among nine sampling locations using Treemix software (Pickrell et al. 2012) to figure out the mechanism of differentiation for *K. punctatus*. The 99.8% variation cutoff inferred by OptM v0.1.5 (Fitak 2021) showed that *m* = 4 was the optimal value (Fig. 4A). Regarding gene flow, our results supported the migration signal from the RZH to the HQ, QHD, and LYG. In addition, there was a gene flow from the ZS to the YQ and NT. As for the Treemix phylogeny, individuals of *K. punctatus* were divided into two main branches, one was the RZH, and the second continued to split into the QHD, DD, SG, LYG, HQ, ZS, as well as the YQ and NT (Fig. 4B).

### Population history dynamics of *K. punctatus*

**Fig. 5:**
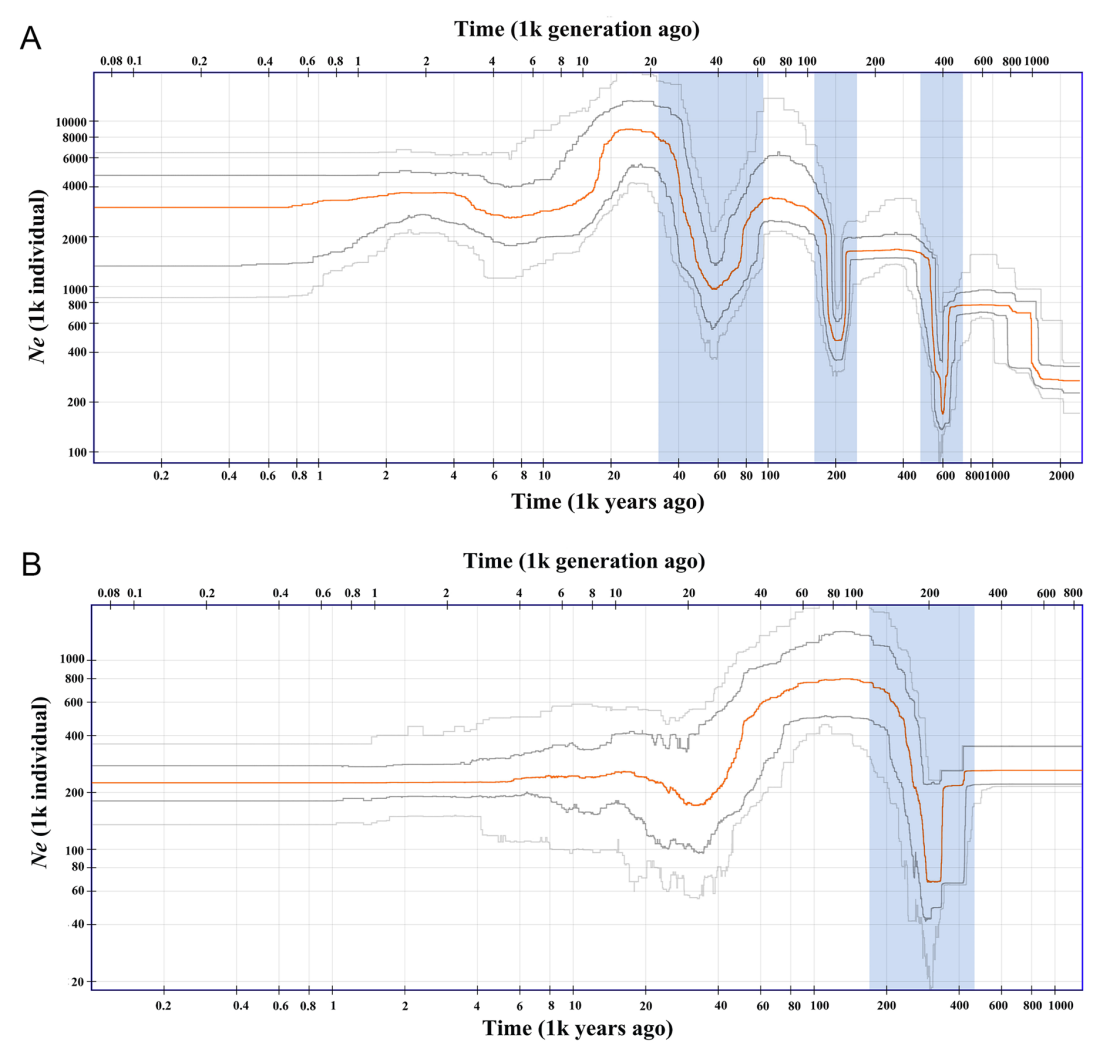
Demographic history of *K. punctatus*. **A** Demographic history of Cluster A. **B** Demographic history of Cluster B. The stairway plot shows the historical effective population size *N*e (*y*-axis) with a generation time of one and a half years. The blue shadows represent population bottlenecks. The red line represents the median of *N*e. Dark gray and light gray lines represent 75 and 95% confidence intervals, respectively

We reconstructed the population history dynamics of Cluster A and Cluster B, respectively to further understand the reasons for differentiation between them. Stairway plot 2 results showed that the first population expansion of Cluster A began nearly 2 million years ago (Mya) (Fig. 5A). Meanwhile, we found that *K. punctatus* of Cluster A had experienced three bottleneck events since then, including 780–500 thousand years ago (Kya), 280–180 Kya and 95–35 Kya bottleneck (Fig. 5A). Then, Cluster A contracted once again around 20 Kya, and the effective population size (*N*e) remained stable (3 × 10^6^) from 5 Kya until recently (Fig. 5A). However, we detected only one bottleneck and one significant population contraction of Cluster B within 1 Mya (Fig. 5B). Cluster B experienced a bottleneck around 420–180 Kya, and contracted again at about 80 Kya. Since 15 Kya, the* N*e of Cluster B was stable at around 2.2 × 10^6^ (Fig. 5B). The above results indicated that the population of *K. punctatus* showed an overall expansion trend, and the population size fluctuated within the Pleistocene ice age (2.6–11.7 Kya).

### Candidate loci and functional genes under putative selection

**Fig. 6:**
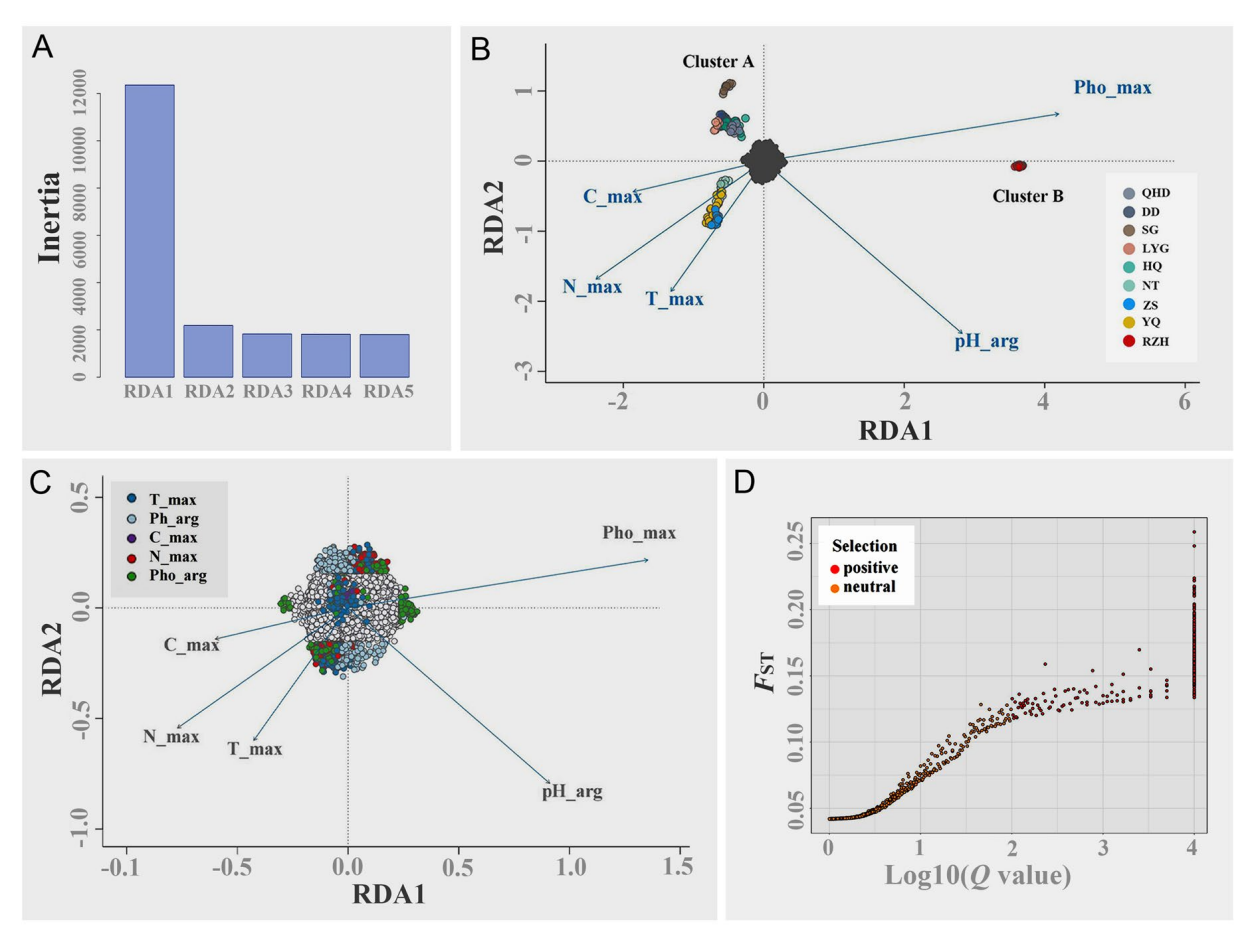
Genomic footprints of adaptation to environmental heterogeneity. **A** The significance of the RDA constraint axis. **B** Redundancy analysis (RDA) on axes 1 and 2 performed with 259,449 SNP loci. The dark grey points at the center of each plot represent the SNPs, and the colored points represent *K. punctatus* individuals of different sampling locations. Blue vectors represent environment variables. **C** Magnification of **B** to highlight SNP loadings on axes 1 and 2. Candidate SNPs are shown as colored points with coding by the most highly correlated environmental variables. SNPs not identified as candidates (neutral SNPs) are shown in light grey. Blue vectors represent environmental variables. **D** Bayesian test for selection performed with 259,449 SNPs using the program BayeScan. Red dots on the right side of the picture are above a 0.99 probability of being candidates of selection. Yellow dots on the left side are neutral SNPs. Chinese populations: Qinhuangdao (QHD), Dandong (DD), Shouguang (SG), Lianyungang (LYG), Nantong (NT), Zhoushan (ZS), and Wenzhou (YQ). Korean population: Jeolla-Do (HQ). Japanese population: Saga (RZH)

Five variables (*T*_max_, pH_avg_, *C*_max_, *N*_max_, and Pho_max_) were retained after removing highly correlated environmental factors based on the Pearson correlation analysis (Supplementary Fig. S1; Supplementary Table S2). Then, the results of the multiple linearity analysis of RDA explained approximately 4.4% of the variance (adjusted *R*^2^ = 0.04402). The RDA1 and RDA2 axes were significant (*P* = 0.001) while the RDA1 axis explained most of the variance, and five environmental factors retained in RDA were also significant as explanatory variables (*P* = 0.001) (Fig. 6A, B). The PCA of the fitted values after multiple linear analysis also showed that 146 *K. punctatus* individuals differentiated into two clusters (Cluster A and Cluster B), and Cluster A had a differentiation between the northern (QHD, DD, SG, LYG and HQ) and southern (NT, ZS and YQ) population (Fig. 6B), which was consistent with the result of NETVIEW (Fig. 3). As shown in Fig. 6B, pH_arg_ and Pho_max_ were significantly correlated with high differentiation between Cluster A and Cluster B, while *C*_max_, *N*_max_ and *T*_max_ were correlated with differentiation among northern and southern populations of *K. punctatus* in Cluster A (Fig. 6B), suggesting that the weak differentiation among individuals in the Cluster A shown in NETVIEW (Fig. 3) are existent and significantly related to the marine environment.

Among all SNPs, a total of 957 candidate loci subject to environmental selection were screened, of which 128 were on the RDA1 axis, 689 on the RDA 2 axis and 143 on the RDA 3 axis (Fig. 6C). Notably, the RDA detected the largest proportion of loci associated with pH_arg_ (281; 29.36%), indicating that pH_arg_ explained more allelic variation than any other environmental variable tested, followed by *T*_max_ (244; 25.50%), then Pho_max_ (240; 25.07%), then was *N*_max_ (183; 19.12%), and finally, *C*_max_ (9; 0.94%) (Fig. 6C). In addition, we screened 309 significant highly differentiated SNPs using BayeScan 2.1 (Fig. 6D). Therefore, we intersected the candidate loci detected by RDA and BayeScan 2.1 and obtained 244 SNP loci associated with local adaptation of *K. punctatus*, of which 78 loci were associated with *N*_max_, 64 loci with pH_arg_, 63 loci with *T*_max_, 36 loci with Pho_max_ and 3 loci with *C*_max_ (Supplementary Table S4).

### Functional annotation of adaptive genes

BEDTools was used to find genes in the 10 kb range upstream and downstream of the adaptive loci, and 56, 53, 45 and 24 genes were detected near the *N*_max_, pH_arg_, *T*_max_ and Pho_max_ association loci, respectively, while none were found near the *C*_max_ association loci (Supplementary Table S5). As shown in Fig. 6C, pH_arg_ association genes were significantly correlated with the differentiation between Cluster A and Cluster B while *T*_max_ association genes correlated with the differentiation between the northern and southern populations of *K. punctatus* in Cluster A. So, Gene ontology (GO) and Kyoto Encyclopedia of Genes and Genomes (KEGG) functional enrichment were only performed for genes associated with pH_arg_ and *T*_max_, respectively. When pH_arg_ association genes were enriched in GO terms, most of them were engaged in molecular functions (MF) such as the epidermal growth factor receptor binding (GO:0005154), the regulation of intramolecular phosphotransferase class activity (GO:0016868) and the regulation of peptidase activity (GO:0061134) (Supplementary Table S6). In addition, there were some genes engaged in a few biological processes (BP) such as the positive regulation of the intrinsic apoptotic signaling pathway (GO:2001244), the negative regulation of centrosome replication (GO:0010826) and the regulation of cytoplasmic mRNA assembly (GO:0010603) (Supplementary Table S6). The most significant 39 GO pathways enriched for *T*_max_ association genes contained 17 cellular components (CC) such as extracellular matrix components (GO:0044420), collagen trimer (GO:0005581) and endoplasmic reticulum lumen (GO:0005788); 22 molecular functions (MF) mainly included binding to extracellular matrix (GO:0050840), protein self-association (GO:0043621), vascular endothelial growth factor receptor activity (GO:0005021), and oxidoreductase activity (GO:0016722), etc. (Supplementary Table S7).

**Fig. 7:**
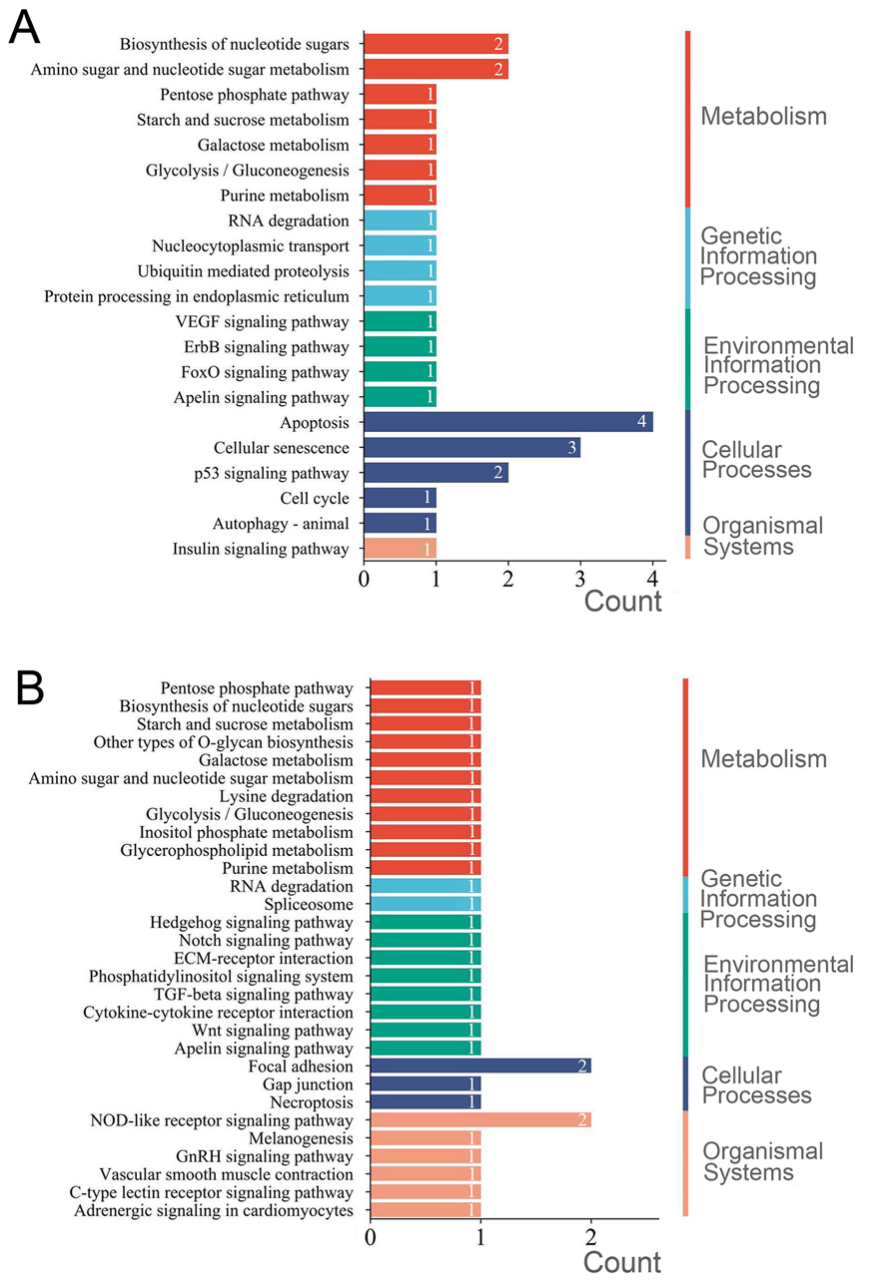
The results of Kyoto Encyclopedia of Genes and Genomes (KEGG) enrichment. **A** KEGG pathway of genes associated with pH_arg_. **B** KEGG pathway of genes associated with *T*_max_

KEGG enrichment results showed that these adaptive genes were mainly engaged in five physiological processes including metabolism, genetic information processing, environmental information processing, cellular processes and organismal systems (Fig. 7). The genes associated with pH_arg_ were enriched to 21 pathways, five of which were related to cellular processes, namely apoptosis, cellular senescence, p53 signaling pathway, cell cycle and autophagy (Fig. 7A). Seven pathways were related to sugar metabolism, including biosynthesis of nucleotide sugars, amino and nucleotide sugar metabolism, pentose phosphate pathway, starch and sucrose metabolism (Fig. 7A). In addition, some pathways related to environmental information processing such as the VEGF signaling pathway, ErbB signaling pathway and FoxO signaling pathway were found as well (Fig. 7A). Among the 30 KEGG pathways enriched for *T*_max_ association genes, there were many pathways related to sugar metabolism as well, but the pathways enriched for the most genes are the cellular focal adhesion and NOD-like receptor signaling pathways (Fig. 7B). In addition, some signaling pathways widely present in vertebrates and invertebrates were identified, such as the Wnt signaling pathway, Hedgehog signaling pathway, and GnRH signaling pathway (Fig. 7B).

Furthermore, we screened eight genes associated with multiple environmental variables from the above genes. Among them, the genes associated with *N*_max_ and pH_arg_ were *KIDINS220* and *SCRIB*; the genes associated with *N*_max_ and *T*_max_ were *VAV3* and *EPB41L3*; the genes associated with pH_arg_ and *T*_max_ were *PAN3* and *PGM5*; the genes associated with Pho_max_ and *T*_max_ were *CCT7* and *HSPA12B* (Table 3). These genes were enriched for GO biological process pathways (*P* < 0.050). Except for *HSPA12B*, all genes were enriched in a variety of GO biological processes, with *SCRIB* enriched in the most pathways (73), followed by *VAV3* (37), then *EPB41L3* (30), *CCT7* and *PGM5* enriched in 18 and 14 pathways, respectively, and *PAN3* and *KIDINS220* enriched in the least pathways, as detailed in Supplementary Table S8.

**Table 3:** List of genes associated with multiple environmental variables

Gene	Description	Name	Environmental variables and *F*_ST_
Kon0109030.1	Kinase D-interacting substrate of 220 kDa	*KIDINS220*	*N*_max_, pH_arg_, *F*_ST_
Kon0177140.1	Scribbled planar cell polarity protein	*SCRIB*	*N*_max_, pH_arg_, *F*_ST_
Kon0176360.1	guanine nucleotide exchange factor	*VAV3*	*N*_max_, *T*_max_, *F*_ST_
Kon0178780.1	Erythrocyte membrane protein band 4.1-like	*EPB41L3*	*N*_max_, *T*_max_, *F*_ST_
Kon0177580.1	PAN2-PAN3 deadenylation complex subunit PAN3	*PAN3*	pH_arg_, *T*_max_, *F*_ST_
Kon0217590.1	Phosphoglucomutase 5	*PGM5*	pH_arg_, *T*_max_, *F*_ST_
Kon0217970.1	Chaperonin containing TCP1 subunit 7	*CCT7*	Pho_max_, *T*_max_, *F*_ST_
Kon0217980.1	Heat shock protein 12B	*HSPA12B*	Pho_max_, *T*_max_, *F*_ST_

## Discussion

In this study, we successfully detected 632,090 SNPs across the genome of *K. punctatus* populations based on RAD-seq for the first time, and analyzed population genetic diversity, population genetic structure and adaptation evolution of *K. punctatus* off the coast of China, Japan, and Korea. The following are the main findings of our study: (1) the population genetic diversity results indicated that all *K. punctatus* sampling locations had high genetic diversity whereas RZH had lower genetic diversity than other locations. (2) The results of pairwise *F*_ST_, Admixture, PCA and NJ tree indicated that 146 *K. punctatus* from nine different locations differentiated into two Clusters (Cluster A and Cluster B), but the NETYIEW plot presented that a small but remarkable genetic differentiation existed between northern and southern population in the Cluster A. (3) Interpopulation gene flow analysis revealed weak gene flow from Cluster B (RZH) to some locations in Cluster A (QHD, HQ and LYG). (4) The results of historical population dynamics showed that Cluster A and Cluster B of *K. punctatus* experienced different times of population contraction and expansion, but generally showed an expansion trend. (5) The RDA plots proved that Cluster A had a differentiation between the northern (QHD, DD, SG, LYG and HQ) and southern (NT, ZS and YQ) population. In addition, the research on the local adaptation of *K. punctatus* found some genes which mediated physiological processes such as growth and development, substance metabolism and immune response of *K. punctatus*. The following is a discussion of the results of this study.

### Population structure and spread history

In this study, we successfully identified genome-wide SNPs using RAD-seq to explore population genetic diversity and population genetic structure of nine sampling locations for *K. punctatus* in China, Japan, and Korea firstly. In terms of the population genetic diversity, the mean values of *H*_E_, *H*_O_ and *π* of nine *K. punctatus* locations based on SNPs were 0.24, 0.23 and 0.25, respectively (Table 1). The RZH exhibited a lower nucleotide diversity than the other eight locations, which was consistent with the results of the proportion of polymorphic loci (Table 1). Since the proportion of polymorphic loci of RZH (59.53%) was significantly lower than the mean value (86.57%) (Table 1), resulting in the low level of nucleotide diversity of RZH, suggesting that the other eight *K. punctatus* locations all generated more genetic variation and had more population resilience during adaptive evolution, showing a more capable of adapting to the changing marine environment than RZH. Furthermore, the level of population genetic diversity for *K. punctatus* in this study was similar to that of some other fishes. For example, Zhang et al. (2016) investigated the population genetic structure and local adaptation of small yellow croaker (*Larimichthys polyactis*) off the coasts of China using RAD-seq, and the population genetic diversity study based on 27,556 SNPs revealed that the mean value of *H*_E_, *H*_O_ and *π* for *L. polyactis* populations were 0.22, 0.23 and 0.22, respectively. In addition, the level of population genetic diversity of some endangered fishes was lower than that in this study. For example, the mean value of *H*_E_, *H*_O_ and *π* for *Anguilla japonica* populations along the Chinese and Japanese coasts were 0.15, 0.16 and 0.15, respectively (Liu 2017), all lower than the values of *K. punctatus* populations in this study. In a word, population genetic diversity results revealed that all *K. punctatus* locations had relatively high genetic diversity except for RZH.

In terms of the population genetic structure, the mean value of *F*_ST_ (0.05) showed a moderate genetic differentiation of *K. punctatus* populations (Meirmans and Hedrick 2011), indicating little variation happened among the *K. punctatus* populations. Overall, only the RZH revealed a high level (*F*_ST_ > 0.15) and significant differentiation in the case of SNPs while other locations of *K. punctatus* showed a low-level differentiation (Table 2), which was like the results of Liu et al. (2020)’s study on *K. punctatus* populations distributed along the coast of China. Furthermore, the results of Admixture, PCA and NJ tree all showed significant differentiation between Cluster A and Cluster B (Fig. 2), which was consistent with previous population genetic studies based on the mtDNA control region (Gwak et al. 2015; Song et al. 2016; Ying 2011). However, the NETVIEW and RDA plot in this study showed a small but significant genetic differentiation between northern and southern population in Cluster A, which was never observed in previous studies based on other genetic markers (Gwak et al. 2015; Song et al. 2016; Ying 2011).

There were three reasons accounting for the population genetic differentiation of *K. punctatus*. (1) The ecological habits of *K. punctatus*. *K. punctatus* has a fixed migratory pattern, as an offshore fish, spawning and nursing almost exclusively in the nearshore and estuaries. However, populations distributed in the Yellow Sea and Bohai Bay have a common overwintering site in the southeastern Yellow Sea. After overwintering, they migrate to shallower waters off the coast of Bohai Bay and Shandong, China for spawning respectively, while populations in other waters do not migrate long distances (Zhang 2001). The relatively independent migratory routes limit the dispersal of southern East China Sea populations of *K. punctatus*, which may lead to the potential differentiation between the northern (QHD, DD, SG, LYG and HQ) and southern (NT, ZS and YQ) population in the Cluster A (Song et al. 2016) (Figs. 3, 6B). However, although the existence of common overwintering sites and the overlap of a few migratory routes may cause the mixing of adjacent *K. punctatus* individuals in Cluster A. The *K. punctatus* in Cluster B only make inshore spawning and overwintering migrations, which may lead to the genetic differentiation between Cluster B and the geographically distant Cluster A due to the long absence of gene exchange (Ying 2011). (2) The influence of ocean currents. Like other fishes, the spawning and hatching grounds of *K. punctatus* tend to be in coastal waters with higher nutrient and productivity levels. But ocean currents may have an impact on the content and distribution of nutrients and productivity in the ocean. For example, there is a branch of Kuroshio warm current between Cluster A and Cluster B (Fig. 1), which makes the chlorophyll A content of the flowing sea area significantly lower than that of the East China Sea shelf and the Japanese coast in spring. The gradual strengthening of the warm current in the summer makes the high productivity only in a small land area along the East China Sea (Fig. 1) (Hamilton et al. 2008). Therefore, the dispersion of spawning and rearing grounds farther offshore is not conducive to the post-migration survival of *K. punctatus*, which may have indirectly led to the gradual differentiation between Cluster A and Cluster B (Ying 2011). (3) Historical climate changes. When Ying (2011) detected the population genetic structure of *K. punctatus* off the coasts of China and Japan based on mtDNA markers, he concluded that the genetic differentiation among them was mainly due to the East China Sea being closed to an inland sea as the drop of sea level during the Last Glacial Maximum (LGM). This has resulted in *K. punctatus* populations distributed in the East China Sea and other seas being isolated in different refuges, promoting differentiation among populations (Wang 1999; Ying 2011). Some marine organisms with similar geographic distribution patterns to *K. punctatus* have also formed similar population genetic structures, such as *CluPea pallasii* (Liu et al. 2007b), *Pennahia argentata* (Han et al. 2008), and *Scapharca broughtonii* (Yokogawa 1997). All population structures of them were affected by the LGM, generating a differentiation among populations along the East China Sea coast and the Japan Sea coast. In addition, Gwak et al. (2015) surveyed the population genetic structure of *K. punctatus* offshore Korea and Japan using fragments of mtDNA and found that two genetic lineages of *K. punctatus* diverged during the Pleistocene glacial cycles. This study hypothesized that the differentiation between the Korean and Japanese populations of *K. punctatus* was caused by the sea level drop during the LGM. At that time, the Yellow Sea almost entirely disappeared and land masses appeared on the seabed of both the Yellow Sea and the southern coast of Korea, leaving the Korean and Japanese coasts separated by a strait-like seaway linking the Northwest Pacific Ocean and the Japan Sea (Park et al. 2006). In our study, Cluster A experienced a population contraction at around 20 Kya, close to the onset of the LGM (~ 22 Kya) (Fig. 5A). It is therefore hypothesized that the shrunk population size of Cluster A was affected by the drop in sea levels during the LGM. Meanwhile, populations in Cluster A were isolated due to the reduced area of the marginal sea (Han and Huang 2008; Yoo et al. 2016), which eventually led to the differentiation between Cluster A and Cluster B.

### Population historical dynamics of *K. punctatus*

The results of the population genetic structure study revealed that there was a high and significant genetic differentiation between Cluster A and Cluster B, so we investigated whether there was gene flow between them using Treemix. The results showed that there was weak gene flow from the RZH to HQ, QHD and LYG, respectively (Fig. 4B), indicating that even though the genetic differentiation among RZH and other locations was significant, potential gene flow existed among them, which was not found in previous studies. In addition, we reconstructed the population history dynamics of Cluster A and Cluster B using Stairway plot 2 and found that Cluster A experienced three distinct bottlenecks in the last two million years (Fig. 5A), while Cluster B experienced only one (Fig. 5B). After the start of the Last Glacial Period (LGP, ~ 110–12 Kya) (Gibbard and Kolfschoten 2005), the population size of both Cluster A and Cluster B changed, the difference being that Cluster A experienced a bottleneck before the population contracted again during the LGM (~ 22 Kya) (Gibbard and Kolfschoten 2005), and then a slight population expansion at the end of the LGP (~ 12 Kya). In contrast, Cluster B experienced only one population contraction at the beginning of the LGP, and the population size gradually stabilized without being greatly affected by the LGM. Overall, there was an expansion trend of *K. punctatus* populations during the Pleistocene ice age. Furthermore, Ying (2011) also detected the expansion trend of the *K. punctatus* population when performing the population genetic analysis based on mitochondrial molecular markers. Combined with historical geographic factors, a series of dramatic climatic fluctuations during the Pleistocene ice age affected various factors of the marine environment, such as seawater temperature and sea level, which can cause changes in the ecological habits and habitat populations of many marine organisms (Liu et al. 2007a). The overall expansion trend of the *K. punctatus* population may be ascribed to the recovery of the marine environment and marine geological changes originally affected by the LGP. As the sea level rise, the isolated sea areas were reconnected, which led to the expansion of the habitat of *K. punctatus*, the increase in population size, and the re-establishment of genetic exchange between populations (Ying 2011). Analogous phenomena have occurred in other fish populations with similar geographic distribution patterns, such as *Lateolabrax japonicus*, *Chelon haematocheilus* and *Lateolabrax maculatus*, all of which have been detected as apparent historical population expansion events after the LGP (Liu et al. 2006a, 2007a). Other marine fishes such as *Engraulis mordax* and *Sardinops sagax* in the Northeast Pacific have also undergone population expansion after the Pleistocene ice age (Lecomte et al. 2004; Liu et al. 2006b).

### The habitat adaptability of *K. punctatus*

In this study, we used RDA analysis to investigate the correlation between local adaptations of *K. punctatus* and marine environmental factors. After combining population genetic data and environmental data for PCA, we found a north–south differentiation among locations of Cluster A, which verified the NETVIEEW plot. However, both Ying (2011) and Liu et al. (2020) failed to detect the genetic structure in the Chinese coastal *K. punctatus* populations based on mitochondrial molecular markers. Therefore, we need to investigate the local adaptive mechanisms of *K. punctatus* populations. Based on RDA, we found that five environmental factors (*T*_max_, pH_arg_, *C*_max_, *N*_max_ and Pho_max_) had a strong impact on the adaptive evolutionary process of *K. punctatus* populations.

As shown in Fig. 6B, pH_arg_ was mainly associated with the genetic differentiation between Cluster A and Cluster B. It has been shown that seawater pH affects behavioral, cellular metabolism, immune response and other processes in marine fishes. For example, the decrease of seawater pH can significantly affect the predation, habitat selection and other behaviors of fishes such as *Amphiprion percula*, *Pomacentrus amboinensis* and *Pomacentrus moluccensis*, reducing their ability to recognize habitats and even changing their temporal rhythm of settlement (Devine et al. 2012; Munday et al. 2009). In addition, the clustering intensity, phototropic behavior, courage level, and the dispersal of marine fishes are all limited by the pH of seawater (Forsgren et al. 2013; Lopes et al. 2016; Maulvault et al. 2018). Meanwhile, we identified several pathways related to glucose metabolism, cellular processes and immune regulation during the KEGG enrichment for pH_arg_ association genes (Fig. 7A). Among them, the p53 signaling pathway is involved in apoptosis, cell growth and differentiation, and DNA damage repair processes (Vousden and Lu 2002). It has been found to be able to mediate the resistance of *L. crocea* bodies to their own visceral nodular disease, suggesting that the p53 signaling pathway also exerts a significant role in the immune regulation of marine fishes (Bai et al. 2022). The p53 signaling pathway is likely to contribute to the adaptation of *K. punctatus* to environmental pH by repairing damage and altering cellular metabolism levels, which indicates that the mean seawater pH can influence various physiological activities in *K. punctatus*, thus affecting the process of adaptive evolution.

*T*_max_ was positively correlated with the differentiation among populations in Cluster A, indicating that temperature was also a major factor influencing the adaptive evolution of *K. punctatus* (Fig. 6B). Previous studies have also shown that seawater temperature can influence the adaptive differentiation of *K. punctatus*. For example, Myoung and Kim (2014) analyzed the population genetic structure of *K. punctatus* along the Korean coasts using mtDNA markers and found that *K. punctatus* populations diverged into two lineages with different sensitivity to temperature, which influenced the local adaptation of *K. punctatus*. Furthermore, it has been shown that temperature may affect the growth, reproduction, and migration of marine fishes, and may drive their adaptive evolution during different life history stages (Johansen and Jones 2011; Pankhurst and Munday 2011). The presence of the GnRH signaling pathway in the KEGG enrichment also supports this conclusion (Fig. 7B), as this signaling pathway can take a vital role in the control of reproductive function, growth and development of fishes (Okubo and Nagahama 2008). Also, Liu et al. (2022a, b) have found that this signaling pathway in male *Opsariichthys bidens* was able to induce the action of growth hormone in males by regulating the secretion of luteinizing hormone and glycoprotein hormone, making males grow faster than females (Liu et al. 2022b). In addition, we identified the Notch signaling pathway that may be directly related to the adaptive evolution of *K. punctatus* (Fig. 7B), which participates in several processes of cell development, including cell morphogenesis and proliferation and differentiation (Skoda et al. 2018; Weng et al. 2004). When Notch receptors bind to ligands, neighboring cells can expand molecular differences between cells by transmitting Notch signals, determining cell fates and ultimately affecting organ formation and morphogenesis (Metz and Bridges 1917). Mutations of loci in the Notch signaling pathway may also cause alterations in biological phenotypes (Mohr 1919), suggesting that it may increase differences among *K. punctatus* populations at both molecular and morphological levels during their adaptation to seawater temperature. Furthermore, even if temperature was not directly determining the local adaptation of *K. punctatus*, other factors closely related to temperature may contribute to the evolution of adaptation. For example, increased water temperatures may reduce the overall oxygen content of the living environment for marine fishes, and less oxygen means less energy to support the physiological performance and movement of marine fishes (Pörtner and Knust 2007), all of which have the potential to affect the environmental adaptation of *K. punctatus*.

Furthermore, we combined RDA and BayeScan 2.1 to screen for highly differentiated environmentally associated genes in *K. punctatus* populations, based on which we identified eight genes associated with multiple environmental variables (Table 3) and enriched them for GO functions (Supplementary Table S8). Among them, the *KIDINS220* gene was mainly engaged in the GO pathways such as the nerve growth factor signaling pathway, the obsolete activation of MAPKK activity, and the cellular response to nerve growth factor stimulus (Supplementary Table S8). The mitogen-activated protein kinase kinase (MAPKK) is engaged in the MAPK signaling pathway, which is essential in regulating cell growth, differentiation, stress adaptation to the environment, inflammatory response and other physiological processes. It had been shown that *KIDINS220* gene expression in zebrafish activated the MAPK signaling pathway (Howe et al. 2013), which had the functions of promoting neuronal cell development and regulating neuronal cell survival in animals (Sutachan et al. 2010; Zhou 2011). Thus, the *KIDINS220* gene may regulate processes, such as cellular responses to environmental stimuli, neurotransmitter receptor transport, or regulate metabolic and immune processes in *K. punctatus* through the MAPK signaling pathway, thus promoting the adaptation of cells or organs to the nitrate content and pH of the marine environment. The protein encoded by the *VAV3* gene can catalyze the interconversion of GDP and GTP and affect the process of intracellular gene transcription (Ulc et al. 2017). Meanwhile, pathways associated with the activation of GTPase activity and the positive regulation of GTPase activity were also identified in GO enrichment (Supplementary Table S8), indicating that the *VAV3* gene can play a role in signaling, material transport, and many cellular activities in which G proteins are involved (Dong and Wen 2005). In addition, GO enrichment revealed that the *VAV3* gene can be involved in 37 biological processes, among which the more important ones for the adaptive evolution of *K. punctatus* were the positive regulation of B cell proliferation, the positive regulation of B cell activation and the involvement of cell surface receptor signaling pathways regulating the immune response in phagocytosis (Supplementary Table S8). B cells are known to produce specific antibodies against invading antigens in vertebrates and have a major role in adaptive immunity (Kincade and Cooper 1971). In addition, B cells can perform phagocytic and bactericidal functions by forming phagocytic lysosomes, which perform a key role in bridging the gap between intrinsic and adaptive immunity in teleost fishes (Wu et al. 2020). The presence of a pathway regulating the involvement of immune response cells in phagocytosis could also corroborate the importance of the *VAV3* gene in the adaptive immunity of *K. punctatus*. Thus, the multiple functions of the protein encoded by the *VAV3* gene are beneficial to the local adaptation of *K. punctatus* to the variable marine environment. The genes associated with pH_arg_ and *T*_max_ were *PAN3* and *PGM5* (Table 3). The protein encoded by the *PAN3* gene is conserved and belonged to the RAS protein family, which may indirectly inhibit excessive apoptosis by suppressing apoptosis protein activity, and inhibit tumor invasion and migration (Yang et al. 2017). Therefore, the *PAN3* gene may enable *K. punctatus* to survive in different seawater pH and water temperatures by inhibiting excessive apoptosis or disease development process. Although the protein encoded by the *PGM5* gene is a muscle structural protein with no enzymatic activity, it has been shown that the *PGM5* gene has a facilitative role in adaptive differentiation between Atlantic herring (*Clupea harengus harengus*) and Baltic herring (*C. harengus membras*) (Gustafsson et al. 2020). In addition, zebrafish embryos have been found to develop severe cardiac and skeletal myopathies with signs of paralysis after knocking out the gene for *PGM5* (Molt et al. 2014). These researches are sufficient to argue that the *PGM5* gene has an important effect on the adaptive evolution and embryonic development of fish. The *PGM5* gene was mainly enriched in the pathways of glucose metabolism and energy metabolism (Supplementary Table S8), suggesting that it also has an impact on physiological processes such as cell metabolism, which may help *K. punctatus* adapt to the marine environment. Furthermore, genes associated with Pho_max_ and *T*_max_ were found (Table 3). The *CCT7* gene encodes the protein which can involve in the regulation of protein localization to chromosome, positive regulation of telomere maintenance, binding of sperm to zona pellucida and positive regulation of DNA biosynthesis (Supplementary Table S8). Surprisingly, ten GO pathways associated with telomeres were enriched. As we know, telomeres have functions in maintaining the lifespan of plant and animal cells, protecting chromosomes, mediating chromosome replication, and regulating cellular senescence, and are closely related to the development of many disease processes. Meanwhile, telomeres have also been found in some long-lived fish to play an active role in the control of the cell cycle, cellular self-phagocytosis and growth regulation, as well as in the regulation of cellular senescence and lifespan (Sauer et al. 2021). Therefore, the *CCT7* gene may be involved in regulating DNA biosynthesis, protein folding and cellular life processes in *K. punctatus*, promoting its adaptation to environmental Pho_max_ and *T*_max_. In addition, we detected a heat shock protein-encoding gene, *HSPA12B*, which is the latest member of the HSP70 protein family (Zhou et al. 2010). The *HSPA12B* gene can participate in the synergistic immunity of animals, which is closely related to angiogenesis and the functional performance of cardiac organs (Zhou et al. 2010). Heat shock proteins are widely present in organisms and play a protective role in the adaptation of organisms to high temperatures. The mean warmest water temperature at the sampling sites of *K. punctatus* populations in this study reached 25–28 °C (Supplementary Table S2). The *HSPA12B* gene may have mobilized the process of synergistic immunity in the body of *K. punctatus* to play a protective role under temperature stress. All these functionally enriched genes detected in highly divergent genomic regions of *K. punctatus* populations may be essential in regulating molecular mechanisms that allow habitat adaptation in *K. punctatus*. Further functional verification of these adaptive genes is needed. In general, the selection signals based on RAD-seq in this study provided a powerful step to understand the potentially adaptive evolution of *K. punctatus*. Furthermore, these findings will help us better protect and exploit the genetic resources of *K. punctatus* and provide a valuable resource for association analysis.

## Conclusions

We successfully employed RAD-seq to explore the population genetic diversity and genetic structure of nine sampling locations for *K. punctatus* in China, Japan, and Korea. The results based on all SNPs revealed significant genetic differentiation between the RZH and other locations. Furthermore, genetic differentiation between northern (QHD, DD, SG, LYG and HQ) and southern (NT, ZS and YQ) population was found. At the same time, we confirmed that the direction of gene flow was from the RZH to HQ, QHD and LYG, and reconstructed the population history dynamics of *K. punctatus*. In addition, we identified outlier SNPs under putative selection and found genes associated with multiple environmental variables that may be crucial in *K. punctatus* adaptation to local habitat heterogeneity. In conclusion, population genetic structure and local habitat adaptation research of *K. punctatus* in this study will provide a strong scientific basis for the conservation and rational use of *K. punctatus* resources. In the future, functional verification of these adaptive genes should be performed to clarify the physiological mechanism of the local adaptation of *K. punctatus* to the marine environment.

### Supplementary Information

Below is the link to the electronic supplementary material.

Supplementary file1 (DOCX 511 KB)

Supplementary file2 (DOCX 107 KB)

## Data Availability

The whole genome of *K. punctatus* at NCBI is under Bioproject accession number PRJNA664710. RAD -seq data of *K. punctatus* is publicly accessible at GenBank under Bioproject accession number PRJNA746100 and Biosample accession numbers SAMN20181051–SAMN20181086, SAMN20181096, SAMN20181098-SAMN20181102, SAMN20181104-SAMN20181168, SAMN20181215- SAMN20181233 and SAMN20181234-SAMN20181254.
