# Supplementary material for: New perspectives on the genetic structure of dotted gizzard shad (Konosirus punctatus) based on RAD-seq
Source: Mar Life Sci Technol. 2024 Feb 12;6(1):50–67. doi: 10.1007/s42995-024-00216-2 (PMC10901767; doi:10.1007/s42995-024-00216-2)
Supplement: Supplementary file 1 — Supplementary file1 (DOCX 511 KB) [file 42995_2024_216_MOESM1_ESM.docx]

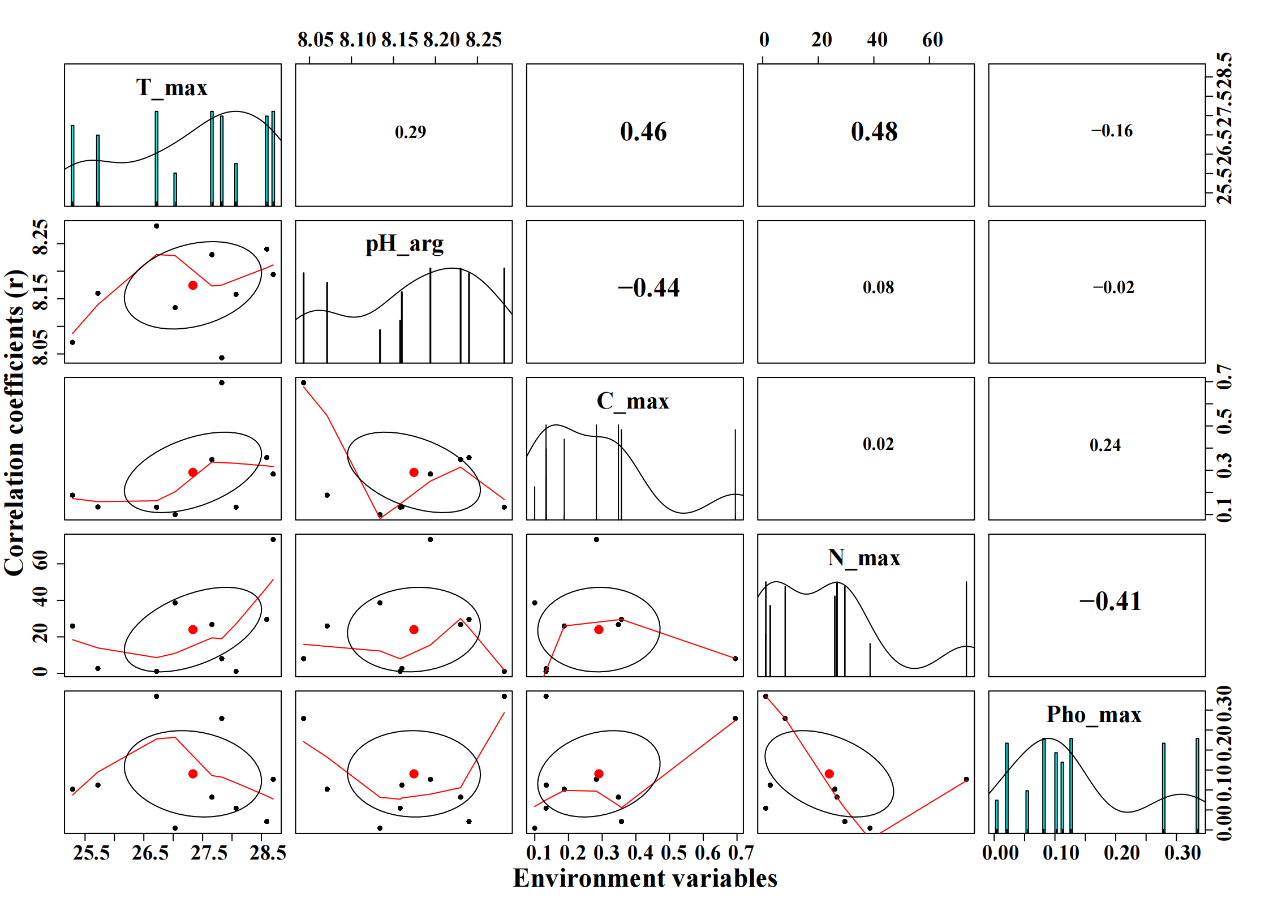


Supplementary Fig. S1. Correlation between environment variables in RDA. Correlation coefficients between environment variables are in the upper right diagonal, with their size scaled to their |r|. The lower left shows scatter plots, while the diagonal shows histograms of the data.


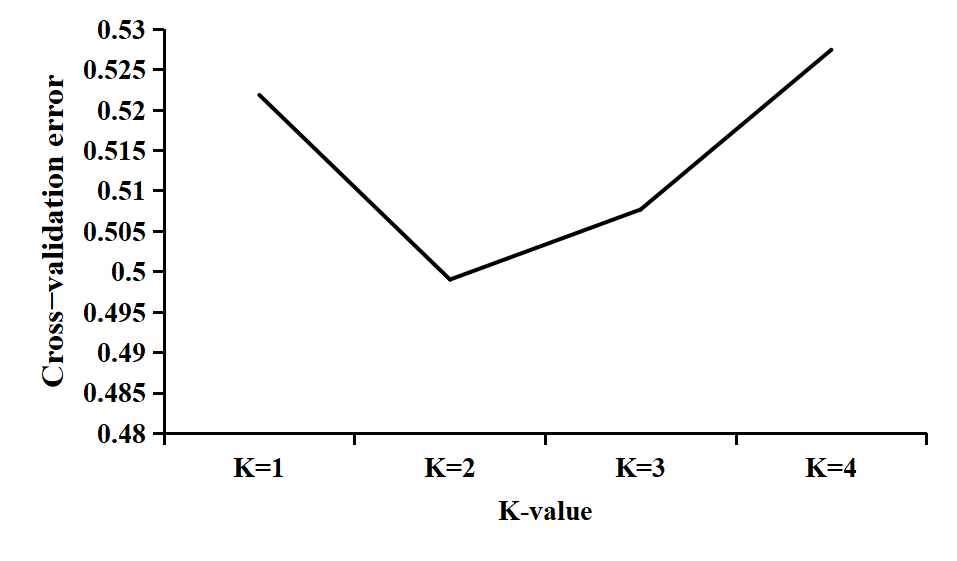


Supplementary Fig. S2. Plots of the *K. punctatus* individuals Admixture clustering for K = 1-4 based on 259,449 SNPs.


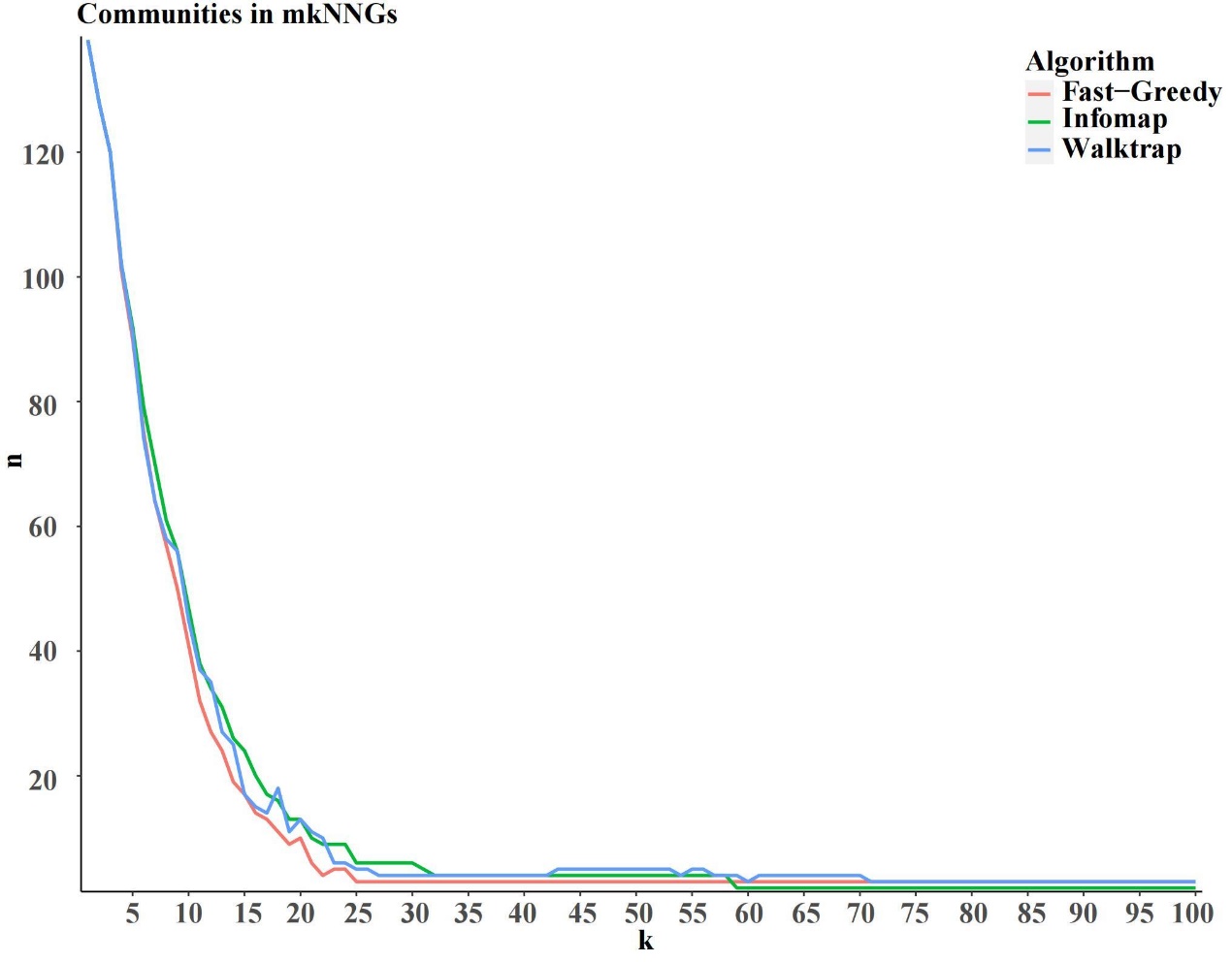


Supplementary Fig. S3. Plots of the *K. punctatus* individuals NETVIEW clustering for K = 1-100 based on 259,449 SNPs. The red line represents the Fast-Greedy algorithm, the green line represents the Infomap algorithm, and the blue line represents the Walktrap algorithm in this figure.
