# Supplementary material for: New perspectives on the genetic structure of dotted gizzard shad (Konosirus punctatus) based on RAD-seq
Source: Mar Life Sci Technol. 2024 Feb 12;6(1):50–67. doi: 10.1007/s42995-024-00216-2 (PMC10901767; doi:10.1007/s42995-024-00216-2)
Supplement: Supplementary file 2 — Supplementary file2 (DOCX 107 KB) [file 42995_2024_216_MOESM2_ESM.docx]

Supplementary Table S1. Sampling details of *K. punctatus*

| Location | Abbreviations | Sample size | Longitude (°E) | Latitude (°N) | Sampling time |
| --- | --- | --- | --- | --- | --- |
| Qinhuangdao | QHD | 15 | 119.66 | 39.92 | 2014.09 |
| Dandong | DD | 17 | 124.22 | 39.82 | 2014.05 |
| Shouguang | SG | 9 | 119.91 | 37.47 | 2020.08 |
| Lianyungang | LYG | 7 | 119.91 | 34.92 | 2018.04 |
| Jeolla-Do | HQ | 19 | 127.11 | 33.99 | 2019.12 |
| Saga | RZH | 20 | 131.96 | 33.62 | 2019.11 |
| Nantong | NT | 20 | 121.7 | 32.1 | 2018.04 |
| Zhoushan | ZS | 20 | 122.78 | 29.96 | 2018.04 |
| Wenzhou | YQ | 19 | 121.30 | 27.9 | 2019.10 |

| Pop ID | T_max | ph_arg | C_max | N_max | Pho_max |
| --- | --- | --- | --- | --- | --- |
| QHD | 25.71882 | 8.16 | 0.13423 | 2.70039 | 0.11213 |
| DD | 25.28776 | 8.071 | 0.18749 | 25.95153 | 0.10186 |
| SG | 28.06799 | 8.158 | 0.13339 | 1.0853 | 0.05436 |
| LYG | 27.03345 | 8.134 | 0.09972 | 38.63528 | 0.00433 |
| HQ | 27.82574 | 8.043 | 0.69506 | 8.07669 | 0.27914 |
| NT | 28.70081 | 8.194 | 0.28288 | 73.30731 | 0.12676 |
| ZS | 27.65836 | 8.23 | 0.3482 | 26.73876 | 0.08201 |
| YQ | 28.59216 | 8.24 | 0.35719 | 29.56182 | 0.02098 |
| RZH | 26.71693 | 8.282 | 0.13341 | 1.12054 | 0.33456 |

Supplementary Table S2. Environmental variables significantly associated with adaptive evolution

Supplementary Table S3. Statistics describing the quality of RAD sequences

| Sample_ID | Raw Reads | Clean Reads | Total_Bases | Error (%) | Q20 (%) | Q30 (%) | GC content (%) | Mapping Rate (%) |
| --- | --- | --- | --- | --- | --- | --- | --- | --- |
| DD1 | 8,127,076 | 8,119,712 | 1,184,730,915 | 0.0257 | 97.68 | 93.57 | 41.96 | 96.93 |
| DD2 | 11,158,486 | 11,151,118 | 1,626,702,397 | 0.0257 | 97.64 | 93.54 | 42 | 97.06 |
| DD3 | 12,707,984 | 12,697,086 | 1,852,419,714 | 0.0256 | 97.73 | 93.7 | 41.95 | 97.11 |
| DD4 | 12,223,590 | 12,215,464 | 1,781,858,373 | 0.0258 | 97.62 | 93.49 | 41.96 | 97.11 |
| DD5 | 10,763,026 | 10,755,426 | 1,569,168,393 | 0.0256 | 97.69 | 93.61 | 41.96 | 97.19 |
| DD6 | 11,690,758 | 11,684,418 | 1,704,622,008 | 0.0257 | 97.69 | 93.6 | 41.95 | 97.18 |
| DD7 | 12,387,326 | 12,380,180 | 1,806,135,897 | 0.0257 | 97.67 | 93.59 | 41.9 | 97.21 |
| DD8 | 12,369,324 | 12,361,162 | 1,803,134,133 | 0.0258 | 97.63 | 93.52 | 42 | 96.59 |
| DD9 | 12,793,386 | 12,781,192 | 1,864,574,342 | 0.0254 | 97.77 | 93.81 | 41.93 | 97.46 |
| DD10 | 12,505,792 | 12,496,902 | 1,823,206,037 | 0.0254 | 97.77 | 93.84 | 41.96 | 97.23 |
| DD11 | 11,836,962 | 11,829,956 | 1,726,111,765 | 0.0255 | 97.74 | 93.74 | 42 | 96.4 |
| DD12 | 7,465,012 | 7,460,876 | 1,088,514,847 | 0.0256 | 97.71 | 93.68 | 41.96 | 97.5 |
| DD13 | 7,778,034 | 7,773,244 | 1,133,904,628 | 0.0259 | 97.59 | 93.35 | 41.98 | 96.99 |
| DD14 | 9,336,836 | 9,330,380 | 1,361,098,963 | 0.0257 | 97.63 | 93.54 | 41.91 | 96.63 |
| DD15 | 6,974,194 | 6,969,080 | 1,016,696,146 | 0.0258 | 97.61 | 93.47 | 41.95 | 97.01 |
| DD16 | 7,535,548 | 7,530,890 | 1,098,818,240 | 0.0255 | 97.73 | 93.73 | 41.91 | 96.18 |
| DD17 | 9,371,722 | 9,363,942 | 1,366,371,000 | 0.0255 | 97.76 | 93.79 | 41.93 | 94.81 |
| HQ1 | 10,565,662 | 10,559,140 | 1,540,815,751 | 0.0259 | 97.58 | 93.33 | 41.91 | 97.32 |
| HQ2 | 10,123,430 | 10,116,874 | 1,476,280,228 | 0.0258 | 97.66 | 93.48 | 41.96 | 97.41 |
| HQ3 | 9,736,920 | 9,730,822 | 1,419,799,692 | 0.026 | 97.56 | 93.29 | 41.96 | 97.5 |
| HQ4 | 9,021,144 | 9,015,706 | 1,315,686,061 | 0.0258 | 97.63 | 93.42 | 41.88 | 97.3 |
| HQ5 | 10,544,690 | 10,538,320 | 1,537,726,922 | 0.0259 | 97.61 | 93.38 | 41.95 | 97.38 |
| HQ6 | 10,788,374 | 10,782,470 | 1,573,235,794 | 0.0259 | 97.58 | 93.34 | 41.95 | 97.44 |
| HQ7 | 10,256,602 | 10,250,290 | 1,495,558,535 | 0.026 | 97.56 | 93.28 | 42.01 | 97.34 |
| HQ8 | 11,360,986 | 11,354,390 | 1,657,141,214 | 0.0256 | 97.73 | 93.66 | 41.95 | 97.39 |
| HQ9 | 9,772,184 | 9,765,474 | 1,425,023,310 | 0.0256 | 97.72 | 93.65 | 42 | 97.47 |
| HQ10 | 9,366,304 | 9,360,710 | 1,365,989,774 | 0.0258 | 97.64 | 93.48 | 41.97 | 97.51 |
| HQ11 | 7,364,226 | 7,359,736 | 1,073,827,682 | 0.0258 | 97.63 | 93.44 | 41.99 | 97.66 |
| HQ12 | 9,674,206 | 9,667,982 | 1,410,484,808 | 0.0261 | 97.52 | 93.15 | 41.96 | 97.33 |
| HQ13 | 8,260,546 | 8,254,986 | 1,204,397,219 | 0.026 | 97.55 | 93.28 | 41.94 | 97.34 |
| HQ14 | 7,995,248 | 7,990,110 | 1,165,782,317 | 0.026 | 97.53 | 93.23 | 41.92 | 97.36 |
| HQ15 | 10,622,472 | 10,615,868 | 1,549,139,991 | 0.0258 | 97.64 | 93.49 | 41.92 | 97.35 |
| HQ16 | 10,254,816 | 10,247,046 | 1,495,395,209 | 0.0257 | 97.67 | 93.55 | 41.85 | 97.6 |
| HQ17 | 8,983,798 | 8,977,394 | 1,309,821,285 | 0.0255 | 97.74 | 93.7 | 41.98 | 97.41 |
| HQ18 | 8,602,880 | 8,597,452 | 1,254,748,257 | 0.0257 | 97.67 | 93.52 | 41.95 | 97.91 |
| HQ19 | 9,745,082 | 9,738,950 | 1,421,213,636 | 0.0257 | 97.65 | 93.51 | 41.96 | 97.41 |
| LYG10 | 10,835,966 | 10,830,436 | 1,580,631,623 | 0.0254 | 97.8 | 93.89 | 41.88 | 97.6 |
| LYG12 | 11,616,680 | 11,609,822 | 1,694,164,862 | 0.0254 | 97.77 | 93.83 | 41.94 | 97.07 |
| LYG13 | 11,300,704 | 11,294,580 | 1,648,309,104 | 0.0253 | 97.82 | 93.91 | 42.03 | 96.91 |
| LYG14 | 11,973,404 | 11,967,310 | 1,746,344,605 | 0.0253 | 97.82 | 93.9 | 41.91 | 96.93 |
| LYG15 | 12,899,020 | 12,892,692 | 1,881,385,322 | 0.0254 | 97.79 | 93.88 | 42.03 | 96.52 |
| LYG16 | 13,685,982 | 13,679,424 | 1,996,161,766 | 0.0254 | 97.79 | 93.86 | 41.9 | 97.22 |
| LYG18 | 12,347,370 | 12,340,974 | 1,801,091,046 | 0.0251 | 97.91 | 94.14 | 41.97 | 97.41 |
| NT1 | 10,757,144 | 10,751,364 | 1,569,178,946 | 0.0252 | 97.85 | 94.01 | 41.96 | 96.68 |
| NT2 | 9,717,690 | 9,712,446 | 1,417,276,317 | 0.0253 | 97.82 | 93.94 | 41.97 | 96.89 |
| NT3 | 11,146,666 | 11,140,976 | 1,625,729,108 | 0.0256 | 97.74 | 93.67 | 41.99 | 96.09 |
| NT4 | 10,990,540 | 10,984,438 | 1,602,824,085 | 0.0254 | 97.77 | 93.84 | 41.94 | 96.75 |
| NT5 | 10,199,144 | 10,192,842 | 1,487,220,374 | 0.0255 | 97.73 | 93.74 | 41.92 | 97.05 |
| NT6 | 11,317,162 | 11,310,832 | 1,650,524,612 | 0.0253 | 97.83 | 93.99 | 41.98 | 97.53 |
| NT7 | 11,652,062 | 11,644,854 | 1,699,194,008 | 0.0252 | 97.86 | 94.03 | 41.95 | 97.35 |
| NT8 | 9,795,870 | 9,790,354 | 1,428,825,820 | 0.0251 | 97.93 | 94.16 | 42 | 97.09 |
| NT9 | 10,752,740 | 10,746,862 | 1,568,280,131 | 0.0252 | 97.85 | 94 | 41.91 | 97.15 |
| NT10 | 10,079,324 | 10,073,618 | 1,470,042,359 | 0.0252 | 97.85 | 94.04 | 41.9 | 97.33 |
| NT11 | 9,749,744 | 9,743,226 | 1,421,825,965 | 0.0252 | 97.88 | 94.09 | 41.91 | 97.49 |
| NT12 | 9,252,024 | 9,247,114 | 1,349,448,972 | 0.0255 | 97.75 | 93.75 | 41.95 | 97.33 |
| NT13 | 9,881,506 | 9,876,236 | 1,441,184,609 | 0.0254 | 97.8 | 93.85 | 41.86 | 97.26 |
| NT14 | 10,859,686 | 10,853,320 | 1,583,740,805 | 0.0255 | 97.73 | 93.73 | 41.91 | 96.55 |
| NT15 | 10,458,040 | 10,451,360 | 1,525,142,802 | 0.0254 | 97.78 | 93.81 | 41.86 | 96.3 |
| NT16 | 9,355,506 | 9,350,128 | 1,364,506,265 | 0.0254 | 97.79 | 93.82 | 41.92 | 96.96 |
| NT17 | 10,168,162 | 10,162,558 | 1,482,708,115 | 0.0255 | 97.73 | 93.74 | 41.92 | 96.67 |
| NT18 | 11,542,400 | 11,536,348 | 1,683,478,010 | 0.0255 | 97.75 | 93.75 | 41.86 | 97.15 |
| NT19 | 10,671,238 | 10,664,320 | 1,556,272,766 | 0.0252 | 97.86 | 94 | 41.87 | 96.75 |
| NT20 | 10,324,406 | 10,317,698 | 1,505,605,056 | 0.0252 | 97.85 | 94 | 41.87 | 97.16 |
| QHD1 | 7,695,702 | 7,689,696 | 1,122,044,574 | 0.0254 | 97.81 | 93.89 | 41.97 | 97.79 |
| QHD2 | 6,538,114 | 6,534,180 | 953,341,239 | 0.0256 | 97.72 | 93.7 | 42.02 | 97.46 |
| QHD3 | 9,319,968 | 9,314,252 | 1,359,103,028 | 0.0255 | 97.73 | 93.76 | 41.94 | 97.76 |
| QHD4 | 6,456,794 | 6,451,090 | 941,168,432 | 0.0255 | 97.75 | 93.78 | 42.01 | 97.14 |
| QHD5 | 7,300,490 | 7,295,730 | 1,064,260,723 | 0.0256 | 97.71 | 93.69 | 42 | 97.58 |
| QHD6 | 7,226,134 | 7,221,534 | 1,053,698,033 | 0.0254 | 97.79 | 93.83 | 41.96 | 97.01 |
| QHD7 | 9,312,974 | 9,306,484 | 1,357,603,805 | 0.0256 | 97.7 | 93.68 | 41.96 | 97.22 |
| QHD8 | 6,431,148 | 6,426,660 | 937,720,162 | 0.0255 | 97.75 | 93.77 | 41.99 | 97.21 |
| QHD9 | 9,347,914 | 9,343,026 | 1,363,298,073 | 0.0255 | 97.76 | 93.77 | 41.91 | 97.57 |
| QHD10 | 10,567,308 | 10,561,460 | 1,540,926,322 | 0.0255 | 97.74 | 93.75 | 41.97 | 97.78 |
| QHD11 | 9,528,584 | 9,523,224 | 1,389,382,256 | 0.0256 | 97.72 | 93.71 | 41.98 | 97.6 |
| QHD12 | 8,667,346 | 8,661,382 | 1,263,706,950 | 0.0253 | 97.83 | 93.96 | 41.99 | 97.42 |
| QHD13 | 8,467,714 | 8,462,510 | 1,234,820,108 | 0.0253 | 97.83 | 93.97 | 41.96 | 97.49 |
| QHD14 | 8,792,324 | 8,787,100 | 1,282,240,323 | 0.0254 | 97.8 | 93.89 | 41.92 | 96.84 |
| QHD15 | 6,469,660 | 6,465,938 | 943,357,398 | 0.0254 | 97.76 | 93.81 | 41.99 | 97.76 |
| RZH1 | 11,434,928 | 11,429,476 | 1,667,836,284 | 0.0254 | 97.78 | 93.84 | 41.99 | 97.87 |
| RZH2 | 11,235,194 | 11,229,580 | 1,638,699,153 | 0.0253 | 97.84 | 93.97 | 41.96 | 97.66 |
| RZH3 | 12,086,828 | 12,080,746 | 1,762,949,443 | 0.0254 | 97.77 | 93.83 | 41.94 | 97.28 |
| RZH4 | 9,806,382 | 9,801,212 | 1,430,396,514 | 0.0254 | 97.81 | 93.89 | 41.93 | 97.3 |
| RZH5 | 10,653,990 | 10,648,690 | 1,554,088,198 | 0.0253 | 97.82 | 93.91 | 41.95 | 97.3 |
| RZH6 | 12,391,986 | 12,386,216 | 1,807,629,539 | 0.0254 | 97.79 | 93.88 | 41.98 | 97.55 |
| RZH7 | 11,180,206 | 11,174,914 | 1,630,882,124 | 0.0254 | 97.79 | 93.86 | 41.95 | 96.94 |
| RZH8 | 10,843,788 | 10,838,618 | 1,582,030,491 | 0.0251 | 97.9 | 94.11 | 41.91 | 96.75 |
| RZH9 | 10,445,072 | 10,439,780 | 1,523,645,011 | 0.0251 | 97.9 | 94.12 | 41.97 | 97.15 |
| RZH10 | 11,356,646 | 11,351,006 | 1,656,456,707 | 0.0252 | 97.84 | 94.01 | 42 | 97.85 |
| RZH11 | 8,577,890 | 8,573,652 | 1,251,133,093 | 0.0253 | 97.82 | 93.94 | 41.93 | 97.38 |
| RZH12 | 15,838,216 | 15,830,200 | 2,310,020,089 | 0.0256 | 97.74 | 93.68 | 41.97 | 97.43 |
| RZH13 | 10,076,108 | 10,070,602 | 1,469,689,322 | 0.0254 | 97.77 | 93.84 | 41.92 | 97.01 |
| RZH14 | 8,744,488 | 8,739,682 | 1,275,256,177 | 0.0255 | 97.74 | 93.77 | 41.98 | 97.47 |
| RZH15 | 10,282,596 | 10,277,210 | 1,499,976,451 | 0.0252 | 97.85 | 94.03 | 41.93 | 97.15 |
| RZH16 | 11,276,890 | 11,270,442 | 1,645,058,697 | 0.0252 | 97.87 | 94.07 | 41.92 | 97.43 |
| RZH17 | 9,249,610 | 9,244,062 | 1,349,246,002 | 0.0251 | 97.93 | 94.17 | 42 | 97.35 |
| RZH18 | 8,483,780 | 8,479,326 | 1,237,681,648 | 0.0252 | 97.86 | 94.02 | 41.93 | 97.13 |
| RZH19 | 10,362,606 | 10,357,148 | 1,511,733,971 | 0.0252 | 97.86 | 94.06 | 41.93 | 97.07 |
| RZH20 | 10,680,694 | 10,675,080 | 1,558,256,543 | 0.0251 | 97.9 | 94.12 | 42.06 | 97.01 |
| SG1 | 8,972,676 | 8,967,974 | 1,308,401,678 | 0.0257 | 97.68 | 93.55 | 41.96 | 97.9 |
| SG2 | 11,413,424 | 11,406,802 | 1,664,164,628 | 0.0256 | 97.7 | 93.69 | 41.91 | 97.73 |
| SG3 | 10,567,826 | 10,562,192 | 1,541,094,809 | 0.0256 | 97.69 | 93.64 | 41.85 | 97.18 |
| SG4 | 13,882,626 | 13,875,218 | 2,024,883,087 | 0.0253 | 97.8 | 93.91 | 41.89 | 97.74 |
| SG5 | 13,465,126 | 13,456,802 | 1,964,508,880 | 0.0252 | 97.87 | 94.05 | 41.92 | 97.79 |
| SG6 | 10,592,482 | 10,586,268 | 1,544,709,853 | 0.0252 | 97.87 | 94.02 | 42.03 | 97.77 |
| SG7 | 12,025,628 | 12,018,980 | 1,753,802,794 | 0.0254 | 97.8 | 93.88 | 41.92 | 97.84 |
| SG8 | 13,173,636 | 13,166,460 | 1,921,299,342 | 0.0253 | 97.8 | 93.92 | 41.92 | 97.88 |
| SG9 | 12,605,686 | 12,598,546 | 1,839,049,005 | 0.0252 | 97.86 | 94.02 | 41.81 | 97.8 |
| YQ1 | 7,348,592 | 7,344,664 | 1,071,924,709 | 0.0252 | 97.87 | 94.07 | 41.99 | 97.61 |
| YQ2 | 7,023,368 | 7,019,780 | 1,024,582,077 | 0.0252 | 97.87 | 94.05 | 41.87 | 97.68 |
| YQ3 | 9,484,066 | 9,479,154 | 1,383,351,113 | 0.0255 | 97.76 | 93.73 | 41.92 | 97.78 |
| YQ4 | 8,322,260 | 8,316,996 | 1,213,735,565 | 0.0254 | 97.79 | 93.9 | 41.89 | 97.73 |
| YQ5 | 7,164,160 | 7,159,630 | 1,044,766,634 | 0.0254 | 97.76 | 93.82 | 41.92 | 97.78 |
| YQ6 | 9,403,200 | 9,398,152 | 1,371,769,789 | 0.0251 | 97.88 | 94.1 | 41.81 | 97.78 |
| YQ7 | 9,271,740 | 9,265,228 | 1,352,347,587 | 0.0251 | 97.91 | 94.15 | 41.91 | 97.66 |
| YQ8 | 8,125,680 | 8,119,584 | 1,185,397,627 | 0.0249 | 97.99 | 94.3 | 41.92 | 97.84 |
| YQ9 | 7,889,816 | 7,885,078 | 1,150,887,260 | 0.0251 | 97.89 | 94.1 | 41.84 | 97.7 |
| YQ10 | 8,619,624 | 8,614,068 | 1,257,239,661 | 0.0251 | 97.89 | 94.12 | 41.91 | 97.74 |
| YQ11 | 8,069,692 | 8,064,704 | 1,177,123,035 | 0.0251 | 97.91 | 94.16 | 41.95 | 97.72 |
| YQ12 | 12,422,022 | 12,414,180 | 1,810,932,847 | 0.0257 | 97.67 | 93.59 | 41.93 | 97.82 |
| YQ13 | 12,347,916 | 12,338,728 | 1,800,054,303 | 0.0255 | 97.73 | 93.7 | 41.94 | 97.74 |
| YQ14 | 13,160,034 | 13,150,930 | 1,918,393,947 | 0.0257 | 97.65 | 93.55 | 41.87 | 97.72 |
| YQ15 | 11,020,254 | 11,013,130 | 1,606,748,265 | 0.0256 | 97.7 | 93.64 | 42 | 97.77 |
| YQ16 | 11,697,900 | 11,690,474 | 1,705,232,109 | 0.0257 | 97.69 | 93.6 | 41.91 | 97.43 |
| YQ17 | 12,059,050 | 12,052,094 | 1,757,949,599 | 0.0257 | 97.67 | 93.58 | 42.01 | 97.86 |
| YQ18 | 12,825,010 | 12,816,386 | 1,869,637,856 | 0.0257 | 97.66 | 93.57 | 41.92 | 97.68 |
| YQ19 | 12,398,742 | 12,390,410 | 1,807,848,107 | 0.0254 | 97.81 | 93.88 | 41.96 | 97.67 |
| ZS1 | 10,323,306 | 10,317,638 | 1,505,773,657 | 0.0253 | 97.81 | 93.9 | 41.87 | 97.2 |
| ZS2 | 8,673,058 | 8,668,182 | 1,264,842,791 | 0.0254 | 97.79 | 93.85 | 41.9 | 97.27 |
| ZS3 | 10,578,450 | 10,572,550 | 1,542,413,647 | 0.0257 | 97.68 | 93.53 | 41.93 | 97.63 |
| ZS4 | 8,750,346 | 8,745,186 | 1,276,026,932 | 0.0256 | 97.72 | 93.71 | 41.9 | 97.03 |
| ZS5 | 8,556,620 | 8,551,834 | 1,247,625,909 | 0.0256 | 97.69 | 93.64 | 41.93 | 97.58 |
| ZS6 | 10,971,842 | 10,965,772 | 1,600,265,766 | 0.0254 | 97.8 | 93.9 | 41.82 | 97.13 |
| ZS7 | 9,895,202 | 9,888,514 | 1,442,997,223 | 0.0253 | 97.82 | 93.94 | 41.92 | 97.28 |
| ZS8 | 8,809,484 | 8,804,136 | 1,284,845,548 | 0.0252 | 97.89 | 94.05 | 41.94 | 97.33 |
| ZS9 | 8,927,056 | 8,921,740 | 1,302,072,043 | 0.0253 | 97.83 | 93.92 | 41.87 | 97.25 |
| ZS10 | 9,974,696 | 9,968,868 | 1,454,646,123 | 0.0253 | 97.81 | 93.92 | 41.89 | 97.24 |
| ZS11 | 8,748,670 | 8,743,036 | 1,275,915,116 | 0.0253 | 97.84 | 93.97 | 41.88 | 97.56 |
| ZS12 | 8,519,618 | 8,515,602 | 1,242,835,469 | 0.0253 | 97.82 | 93.93 | 42 | 97.41 |
| ZS13 | 8,475,820 | 8,471,272 | 1,236,447,661 | 0.0252 | 97.88 | 94.06 | 41.94 | 97.22 |
| ZS14 | 8,785,286 | 8,780,802 | 1,281,612,188 | 0.0253 | 97.8 | 93.91 | 41.93 | 96.83 |
| ZS15 | 8,656,072 | 8,651,638 | 1,262,824,713 | 0.0252 | 97.85 | 93.99 | 41.91 | 97.38 |
| ZS16 | 8,307,498 | 8,303,444 | 1,211,828,677 | 0.0253 | 97.84 | 93.97 | 41.9 | 97.46 |
| ZS17 | 8,327,568 | 8,323,800 | 1,214,830,638 | 0.0253 | 97.82 | 93.95 | 41.92 | 97.18 |
| ZS18 | 8,794,288 | 8,789,644 | 1,282,683,318 | 0.0254 | 97.8 | 93.89 | 41.91 | 97.65 |
| ZS19 | 8,938,030 | 8,933,670 | 1,303,836,535 | 0.0251 | 97.92 | 94.15 | 41.96 | 97.36 |
| ZS20 | 8,621,984 | 8,617,578 | 1,257,827,598 | 0.025 | 97.93 | 94.19 | 41.92 | 97.24 |

Supplementary Table S4. Highly differentiated environmental adaptive loci

| SNP | *F*_ST_ | Environmental variables | Correlation |
| --- | --- | --- | --- |
| chr1.4279563 | 0.21186 | T_max | 0.607411305 |
| chr10.282515 | 0.14647 | Pho_arg | 0.365945502 |
| chr10.3370041 | 0.13167 | Pho_arg | 0.532369552 |
| chr10.4978840 | 0.15513 | N_max | 0.34943749 |
| chr10.6212597 | 0.16852 | Pho_arg | 0.381799998 |
| chr10.8362301 | 0.15608 | N_max | 0.332696462 |
| chr10.8362314 | 0.14322 | Ph_arg | 0.475677455 |
| chr11.11105953 | 0.1954 | N_max | 0.331175822 |
| chr11.27029816 | 0.16555 | Pho_arg | 0.522077944 |
| chr11.27067690 | 0.17744 | Ph_arg | 0.448944256 |
| chr11.27107595 | 0.1473 | Ph_arg | 0.504224978 |
| chr11.28346673 | 0.15887 | N_max | 0.44667278 |
| chr11.28912632 | 0.18984 | N_max | 0.388305484 |
| chr11.29388784 | 0.22184 | N_max | 0.516416287 |
| chr11.30028529 | 0.13866 | Pho_arg | 0.472507305 |
| chr11.30237026 | 0.21066 | Pho_arg | 0.487335978 |
| chr11.30282306 | 0.13284 | Ph_arg | 0.390918283 |
| chr11.30327105 | 0.15886 | T_max | 0.43177378 |
| chr11.30584951 | 0.1551 | Pho_arg | 0.54224603 |
| chr11.30710954 | 0.12928 | N_max | 0.319828393 |
| chr11.30767852 | 0.19739 | N_max | 0.458372864 |
| chr11.30826084 | 0.14293 | N_max | 0.473251113 |
| chr11.30850570 | 0.20422 | N_max | 0.439712276 |
| chr11.31095707 | 0.15139 | N_max | 0.501288069 |
| chr11.31289848 | 0.15138 | N_max | 0.451115776 |
| chr12.5609626 | 0.22381 | C_max | 0.588022303 |
| chr15.15089909 | 0.13118 | Pho_arg | 0.346708491 |
| chr17.11345684 | 0.18064 | T_max | 0.34615854 |
| chr17.11524592 | 0.16233 | T_max | 0.432112497 |
| chr17.11742661 | 0.142 | N_max | 0.323874321 |
| chr17.11831967 | 0.17585 | T_max | 0.350524121 |
| chr17.11926449 | 0.1694 | Ph_arg | 0.356230274 |
| chr17.12008298 | 0.16081 | Ph_arg | 0.304272884 |
| chr17.12092337 | 0.13238 | Ph_arg | 0.2011604 |
| chr17.12218752 | 0.18806 | N_max | 0.360902268 |
| chr17.12263870 | 0.18974 | N_max | 0.364147367 |
| chr17.12348712 | 0.19135 | N_max | 0.311516519 |
| chr17.12453840 | 0.1397 | N_max | 0.38312398 |
| chr17.12507925 | 0.18606 | Ph_arg | 0.35090074 |
| chr17.12609388 | 0.17178 | N_max | 0.41794515 |
| chr17.12616177 | 0.19647 | Ph_arg | 0.366303005 |
| chr17.12736300 | 0.17208 | Ph_arg | 0.320342211 |
| chr17.12811501 | 0.15677 | N_max | 0.297652982 |
| chr17.13183166 | 0.1335 | T_max | 0.317178261 |
| chr17.13546794 | 0.1741 | Ph_arg | 0.392009332 |
| chr17.13622078 | 0.17187 | N_max | 0.368575525 |
| chr17.13741626 | 0.1933 | Ph_arg | 0.390512622 |
| chr17.13857932 | 0.2025 | Pho_arg | 0.444503636 |
| chr17.13891510 | 0.20316 | T_max | 0.37238264 |
| chr17.13926785 | 0.15245 | N_max | 0.402389273 |
| chr17.13964094 | 0.14599 | N_max | 0.409117808 |
| chr17.14058979 | 0.15603 | T_max | 0.343932861 |
| chr17.14115395 | 0.17391 | T_max | 0.336842556 |
| chr17.14204237 | 0.21289 | Ph_arg | 0.414713799 |
| chr17.14252250 | 0.18397 | T_max | 0.328776207 |
| chr17.14282223 | 0.19795 | T_max | 0.415976815 |
| chr17.14373135 | 0.17489 | Ph_arg | 0.472219804 |
| chr17.14382853 | 0.15552 | Pho_arg | 0.317691941 |
| chr17.14440017 | 0.19032 | N_max | 0.394974603 |
| chr17.14480660 | 0.15607 | Ph_arg | 0.34818139 |
| chr17.14589368 | 0.18378 | T_max | 0.418407954 |
| chr17.14801187 | 0.13276 | N_max | 0.404497384 |
| chr17.14863220 | 0.1821 | T_max | 0.388304827 |
| chr17.15025974 | 0.17204 | T_max | 0.348617924 |
| chr17.15195804 | 0.14687 | T_max | 0.379732959 |
| chr17.15310909 | 0.17134 | Ph_arg | 0.268569514 |
| chr17.15413645 | 0.16292 | N_max | 0.33929744 |
| chr17.15481050 | 0.16213 | T_max | 0.338464036 |
| chr17.15839506 | 0.20119 | N_max | 0.485943073 |
| chr17.15940214 | 0.15614 | N_max | 0.446589 |
| chr17.15984430 | 0.17902 | Ph_arg | 0.345954269 |
| chr17.16031419 | 0.16832 | Ph_arg | 0.341497364 |
| chr17.16058219 | 0.14093 | N_max | 0.328337341 |
| chr17.16188836 | 0.17072 | N_max | 0.420775472 |
| chr17.16314035 | 0.15169 | Ph_arg | 0.326979254 |
| chr17.16555212 | 0.18479 | N_max | 0.445814056 |
| chr17.16601819 | 0.16002 | T_max | 0.33207547 |
| chr17.16740168 | 0.21695 | T_max | 0.329504706 |
| chr17.16957165 | 0.14404 | T_max | 0.413603544 |
| chr17.17115165 | 0.13752 | N_max | 0.287358439 |
| chr17.17190939 | 0.15192 | Pho_arg | 0.424951923 |
| chr17.17279372 | 0.16144 | N_max | 0.259590001 |
| chr17.17354347 | 0.20015 | Ph_arg | 0.415839138 |
| chr17.17495189 | 0.16011 | N_max | 0.39616689 |
| chr17.17589765 | 0.16321 | Ph_arg | 0.31046233 |
| chr17.17620472 | 0.17216 | N_max | 0.404157416 |
| chr17.17715896 | 0.14705 | T_max | 0.286677369 |
| chr17.17790043 | 0.1931 | Ph_arg | 0.453143596 |
| chr17.17901031 | 0.14643 | Pho_arg | 0.343008701 |
| chr17.17942259 | 0.17029 | T_max | 0.368338627 |
| chr17.18197662 | 0.17309 | T_max | 0.393893529 |
| chr17.18237448 | 0.16707 | T_max | 0.407588478 |
| chr17.18397775 | 0.15376 | T_max | 0.340988205 |
| chr17.18523737 | 0.14663 | T_max | 0.312307952 |
| chr17.18557264 | 0.15318 | Pho_arg | 0.314800001 |
| chr17.18588703 | 0.1778 | T_max | 0.37099442 |
| chr17.18614220 | 0.14608 | Pho_arg | 0.325035827 |
| chr17.18714805 | 0.1603 | Ph_arg | 0.387242831 |
| chr17.18850716 | 0.15317 | N_max | 0.27888397 |
| chr17.18994244 | 0.16392 | T_max | 0.3968646 |
| chr17.19026293 | 0.19621 | Ph_arg | 0.262208935 |
| chr17.19080623 | 0.18233 | Ph_arg | 0.4812865 |
| chr17.19231875 | 0.16234 | N_max | 0.26168106 |
| chr17.19298055 | 0.12601 | Pho_arg | 0.468304182 |
| chr17.19327911 | 0.17693 | T_max | 0.306797959 |
| chr17.19342003 | 0.15181 | Ph_arg | 0.486544672 |
| chr17.19476652 | 0.14241 | Ph_arg | 0.468979593 |
| chr17.19598449 | 0.21323 | T_max | 0.428196412 |
| chr17.19712114 | 0.13026 | T_max | 0.387102284 |
| chr17.19745020 | 0.13822 | Ph_arg | 0.414789157 |
| chr17.19804197 | 0.19172 | N_max | 0.377325099 |
| chr17.19877493 | 0.17861 | T_max | 0.411599608 |
| chr17.19925040 | 0.15253 | N_max | 0.30691627 |
| chr17.19948711 | 0.15969 | Ph_arg | 0.281184806 |
| chr17.20050545 | 0.14852 | T_max | 0.306740121 |
| chr17.20234860 | 0.25871 | T_max | 0.4738505 |
| chr17.20286646 | 0.18551 | N_max | 0.372907443 |
| chr17.20390843 | 0.14879 | N_max | 0.246067102 |
| chr17.20474356 | 0.15066 | N_max | 0.405837038 |
| chr17.20527472 | 0.14936 | Ph_arg | 0.419844518 |
| chr17.20726439 | 0.18795 | T_max | 0.386869532 |
| chr17.20827873 | 0.18118 | N_max | 0.37717065 |
| chr17.20862802 | 0.19711 | T_max | 0.41300366 |
| chr17.21045453 | 0.14731 | Pho_arg | 0.309022108 |
| chr17.21187238 | 0.19431 | T_max | 0.332737115 |
| chr17.21281474 | 0.16979 | N_max | 0.40856083 |
| chr17.21369058 | 0.18372 | Ph_arg | 0.428254744 |
| chr17.21404700 | 0.16947 | T_max | 0.33700154 |
| chr17.21451916 | 0.16158 | Pho_arg | 0.363371061 |
| chr17.21515365 | 0.187 | N_max | 0.410472421 |
| chr17.21662245 | 0.18347 | T_max | 0.410961356 |
| chr17.21832329 | 0.15272 | N_max | 0.425841994 |
| chr17.21945811 | 0.17622 | N_max | 0.372304594 |
| chr17.22037873 | 0.15338 | Ph_arg | 0.358555027 |
| chr17.22049399 | 0.16832 | N_max | 0.456745802 |
| chr17.22094416 | 0.14752 | N_max | 0.28835808 |
| chr17.22129693 | 0.15158 | N_max | 0.406347539 |
| chr17.22268082 | 0.15805 | T_max | 0.362724987 |
| chr17.22374743 | 0.1426 | T_max | 0.289950906 |
| chr17.22393408 | 0.15086 | N_max | 0.450586575 |
| chr17.22465998 | 0.13565 | Pho_arg | 0.373361854 |
| chr17.22565094 | 0.2177 | T_max | 0.457813778 |
| chr17.227550 | 0.13613 | N_max | 0.510318703 |
| chr17.22760651 | 0.16503 | N_max | 0.326427246 |
| chr17.22830214 | 0.18378 | Ph_arg | 0.486991121 |
| chr17.22885921 | 0.17406 | N_max | 0.386655999 |
| chr17.23004074 | 0.13859 | Ph_arg | 0.325468999 |
| chr17.23007426 | 0.18433 | N_max | 0.366495822 |
| chr17.23149614 | 0.18133 | N_max | 0.426164363 |
| chr17.23154637 | 0.15052 | T_max | 0.283646885 |
| chr17.23217543 | 0.1577 | N_max | 0.390035616 |
| chr17.23299930 | 0.16126 | N_max | 0.317867746 |
| chr17.23487476 | 0.129 | N_max | 0.383602669 |
| chr17.23560281 | 0.15541 | Ph_arg | 0.284712364 |
| chr17.23660782 | 0.14372 | T_max | 0.354712988 |
| chr17.23743463 | 0.16041 | Ph_arg | 0.333981076 |
| chr17.23769619 | 0.15184 | T_max | 0.365165439 |
| chr17.23806251 | 0.17544 | Pho_arg | 0.445366712 |
| chr17.23892100 | 0.14736 | Ph_arg | 0.245419066 |
| chr17.23993489 | 0.17458 | N_max | 0.476109234 |
| chr17.24031990 | 0.13505 | Ph_arg | 0.339226791 |
| chr17.24120579 | 0.15092 | Pho_arg | 0.371297543 |
| chr17.24146879 | 0.14012 | N_max | 0.276134086 |
| chr17.24226741 | 0.17165 | T_max | 0.388067635 |
| chr17.24306951 | 0.15457 | T_max | 0.31863907 |
| chr17.24431277 | 0.1466 | N_max | 0.356535554 |
| chr17.251319 | 0.16569 | Pho_arg | 0.404184449 |
| chr17.453370 | 0.21642 | T_max | 0.441215774 |
| chr2.26241579 | 0.14573 | T_max | 0.33584056 |
| chr2.26574520 | 0.14697 | N_max | 0.384661129 |
| chr2.26985303 | 0.17361 | C_max | 0.35580864 |
| chr2.27156648 | 0.18047 | N_max | 0.403462666 |
| chr2.27236308 | 0.18083 | N_max | 0.409873429 |
| chr2.27318663 | 0.17603 | Pho_arg | 0.412526521 |
| chr2.27391718 | 0.17355 | Pho_arg | 0.400340153 |
| chr2.27624997 | 0.13655 | N_max | 0.407427926 |
| chr2.27697344 | 0.17948 | N_max | 0.381173979 |
| chr2.27981381 | 0.12789 | Pho_arg | 0.343069714 |
| chr2.28219945 | 0.18009 | N_max | 0.425675899 |
| chr2.28291792 | 0.1572 | Pho_arg | 0.382558411 |
| chr2.28449349 | 0.16395 | N_max | 0.401634583 |
| chr2.28521883 | 0.1337 | N_max | 0.392773664 |
| chr2.28568657 | 0.17685 | N_max | 0.401921263 |
| chr2.28643224 | 0.15067 | N_max | 0.40906511 |
| chr2.28677204 | 0.15206 | Pho_arg | 0.366223723 |
| chr21.16448574 | 0.24812 | T_max | 0.557379161 |
| chr22.10504622 | 0.14574 | T_max | 0.283734605 |
| chr22.10536826 | 0.15533 | Ph_arg | 0.408537303 |
| chr22.11428940 | 0.15657 | Ph_arg | 0.340749598 |
| chr22.11534883 | 0.16297 | Ph_arg | 0.397116246 |
| chr22.11555012 | 0.21031 | Pho_arg | 0.435380243 |
| chr22.11796237 | 0.15933 | N_max | 0.352366885 |
| chr22.11913586 | 0.15517 | Ph_arg | 0.322649652 |
| chr22.11967435 | 0.12953 | Ph_arg | 0.381500373 |
| chr22.12365929 | 0.12939 | Ph_arg | 0.417989563 |
| chr22.12436558 | 0.1254 | Pho_arg | 0.295921499 |
| chr22.12685151 | 0.16762 | Ph_arg | 0.31017513 |
| chr22.12739289 | 0.16 | N_max | 0.424565171 |
| chr22.13309119 | 0.14608 | N_max | 0.405419905 |
| chr22.13883130 | 0.14343 | Ph_arg | 0.425227857 |
| chr22.14037579 | 0.15343 | T_max | 0.362519843 |
| chr22.14235857 | 0.14115 | Pho_arg | 0.326718452 |
| chr22.14315015 | 0.18544 | T_max | 0.351328503 |
| chr22.14451570 | 0.13482 | Ph_arg | 0.487993801 |
| chr22.14598366 | 0.15073 | Ph_arg | 0.378741653 |
| chr22.14674137 | 0.12726 | Ph_arg | 0.408632592 |
| chr22.14709264 | 0.15082 | T_max | 0.367765485 |
| chr22.14751037 | 0.1481 | T_max | 0.25348256 |
| chr22.14786791 | 0.14595 | Ph_arg | 0.283148615 |
| chr22.14898069 | 0.13019 | Pho_arg | 0.397698169 |
| chr22.14991105 | 0.16894 | Ph_arg | 0.304335308 |
| chr22.15036735 | 0.13002 | Ph_arg | 0.341117252 |
| chr22.15120006 | 0.15076 | Pho_arg | 0.436975853 |
| chr22.15203354 | 0.16623 | Pho_arg | 0.241579714 |
| chr22.15240583 | 0.15955 | Pho_arg | 0.377730937 |
| chr22.15383372 | 0.14348 | Ph_arg | 0.416471659 |
| chr22.15517002 | 0.15126 | Ph_arg | 0.407578574 |
| chr22.15547451 | 0.18905 | Ph_arg | 0.449052414 |
| chr22.15644288 | 0.19361 | T_max | 0.377894764 |
| chr22.15778902 | 0.17404 | Ph_arg | 0.309160228 |
| chr22.15826036 | 0.16279 | Ph_arg | 0.351216515 |
| chr22.16038457 | 0.15389 | Pho_arg | 0.39226426 |
| chr22.7455059 | 0.16638 | Ph_arg | 0.52891234 |
| chr22.7704941 | 0.16489 | N_max | 0.464450742 |
| chr22.7947203 | 0.16725 | T_max | 0.397368018 |
| chr22.8215084 | 0.13126 | Pho_arg | 0.329387624 |
| chr22.8273528 | 0.15661 | T_max | 0.426523428 |
| chr22.8414271 | 0.13779 | T_max | 0.377343134 |
| chr22.8492684 | 0.15626 | Ph_arg | 0.369679188 |
| chr22.8635755 | 0.16241 | T_max | 0.388287512 |
| chr22.8838838 | 0.15002 | Ph_arg | 0.551226676 |
| chr22.8890807 | 0.15431 | T_max | 0.329946414 |
| chr22.8943430 | 0.14423 | Ph_arg | 0.53171273 |
| chr22.8966209 | 0.13982 | T_max | 0.424771943 |
| chr22.9041451 | 0.15055 | Ph_arg | 0.554203527 |
| chr22.9239840 | 0.13316 | Ph_arg | 0.251385145 |
| chr22.9345287 | 0.1385 | N_max | 0.325658955 |
| chr22.9708729 | 0.14517 | T_max | 0.355560972 |
| chr22.9858573 | 0.14134 | Pho_arg | 0.489633017 |
| chr22.9858762 | 0.17851 | T_max | 0.358630633 |
| chr22.9908882 | 0.12968 | Ph_arg | 0.557726107 |
| chr4.5924208 | 0.15284 | N_max | 0.347325777 |
| chr5.21251171 | 0.15395 | N_max | 0.225521811 |
| chr6.29039091 | 0.22226 | C_max | 0.74124121 |

Supplementary Table S5. List of adaptive genes

| Gene | Environmental variables | Gene | Environmental variables |
| --- | --- | --- | --- |
| Kon0109030.1 | N_max | Kon0218520.1 | pH_arg |
| Kon0120230.1 | N_max | Kon0218530.1 | pH_arg |
| Kon0126230.1 | N_max | Kon0218790.1 | pH_arg |
| Kon0126380.1 | N_max | Kon0218820.1 | pH_arg |
| Kon0126830.1 | N_max | Kon0219170.1 | pH_arg |
| Kon0126990.1 | N_max | Kon0219420.1 | pH_arg |
| Kon0175390.1 | N_max | Kon0219430.1 | pH_arg |
| Kon0175520.1 | N_max | Kon0219440.1 | pH_arg |
| Kon0175530.1 | N_max | Kon0219680.1 | pH_arg |
| Kon0175660.1 | N_max | Kon0219760.1 | pH_arg |
| Kon0176020.1 | N_max | Kon0219810.1 | pH_arg |
| Kon0176150.1 | N_max | Kon0219890.1 | pH_arg |
| Kon0176160.1 | N_max | Kon0219960.1 | pH_arg |
| Kon0176180.1 | N_max | Kon0219970.1 | pH_arg |
| Kon0176360.1 | N_max | Kon0220220.1 | pH_arg |
| Kon0176490.1 | N_max | Kon0220240.1 | pH_arg |
| Kon0176610.1 | N_max | Kon0220270.1 | pH_arg |
| Kon0176640.1 | N_max | Kon0220280.1 | pH_arg |
| Kon0176920.1 | N_max | Kon0220340.1 | pH_arg |
| Kon0177070.1 | N_max | Kon0220360.1 | pH_arg |
| Kon0177140.1 | N_max | Kon0001120.1 | T_max |
| Kon0177470.1 | N_max | Kon0126680.1 | T_max |
| Kon0177480.1 | N_max | Kon0172770.1 | T_max |
| Kon0177760.1 | N_max | Kon0175260.1 | T_max |
| Kon0177830.1 | N_max | Kon0175480.1 | T_max |
| Kon0177860.1 | N_max | Kon0175780.1 | T_max |
| Kon0178090.1 | N_max | Kon0176130.1 | T_max |
| Kon0178190.1 | N_max | Kon0176190.1 | T_max |
| Kon0178330.1 | N_max | Kon0176230.1 | T_max |
| Kon0178340.1 | N_max | Kon0176360.1 | T_max |
| Kon0178410.1 | N_max | Kon0176420.1 | T_max |
| Kon0178450.1 | N_max | Kon0176770.1 | T_max |
| Kon0178460.1 | N_max | Kon0176810.1 | T_max |
| Kon0178670.1 | N_max | Kon0176860.1 | T_max |
| Kon0178780.1 | N_max | Kon0177170.1 | T_max |
| Kon0178800.1 | N_max | Kon0177210.1 | T_max |
| Kon0178970.1 | N_max | Kon0177250.1 | T_max |
| Kon0178990.1 | N_max | Kon0177300.1 | T_max |
| Kon0021850.1 | N_max | Kon0177310.1 | T_max |
| Kon0022070.1 | N_max | Kon0177320.1 | T_max |
| Kon0022240.1 | N_max | Kon0177390.1 | T_max |
| Kon0022250.1 | N_max | Kon0177570.1 | T_max |
| Kon0022260.1 | N_max | Kon0177580.1 | T_max |
| Kon0022270.1 | N_max | Kon0177790.1 | T_max |
| Kon0022280.1 | N_max | Kon0177930.1 | T_max |
| Kon0022500.1 | N_max | Kon0178040.1 | T_max |
| Kon0022560.1 | N_max | Kon0178170.1 | T_max |
| Kon0022570.1 | N_max | Kon0178260.1 | T_max |
| Kon0022620.1 | N_max | Kon0178550.1 | T_max |
| Kon0022660.1 | N_max | Kon0178560.1 | T_max |
| Kon0022670.1 | N_max | Kon0178780.1 | T_max |
| Kon0022690.1 | N_max | Kon0178860.1 | T_max |
| Kon0217850.1 | N_max | Kon0178900.1 | T_max |
| Kon0218740.1 | N_max | Kon0021730.1 | T_max |
| Kon0219190.1 | N_max | Kon0217340.1 | T_max |
| Kon0058640.1 | N_max | Kon0217490.1 | T_max |
| Kon0109030.1 | pH_arg | Kon0217560.1 | T_max |
| Kon0125870.1 | pH_arg | Kon0217590.1 | T_max |
| Kon0125880.1 | pH_arg | Kon0217940.1 | T_max |
| Kon0175500.1 | pH_arg | Kon0217970.1 | T_max |
| Kon0175610.1 | pH_arg | Kon0217980.1 | T_max |
| Kon0175990.1 | pH_arg | Kon0219520.1 | T_max |
| Kon0176220.1 | pH_arg | Kon0219530.1 | T_max |
| Kon0176430.1 | pH_arg | Kon0219830.1 | T_max |
| Kon0176940.1 | pH_arg | Kon0220310.1 | T_max |
| Kon0177140.1 | pH_arg | Kon0106630.1 | Pho_max |
| Kon0177400.1 | pH_arg | Kon0107680.1 | Pho_max |
| Kon0177580.1 | pH_arg | Kon0126780.1 | Pho_max |
| Kon0177730.1 | pH_arg | Kon0126790.1 | Pho_max |
| Kon0177800.1 | pH_arg | Kon0160540.1 | Pho_max |
| Kon0177890.1 | pH_arg | Kon0160550.1 | Pho_max |
| Kon0178130.1 | pH_arg | Kon0176110.1 | Pho_max |
| Kon0178140.1 | pH_arg | Kon0177340.1 | Pho_max |
| Kon0178150.1 | pH_arg | Kon0177540.1 | Pho_max |
| Kon0178390.1 | pH_arg | Kon0177550.1 | Pho_max |
| Kon0178400.1 | pH_arg | Kon0178180.1 | Pho_max |
| Kon0178880.1 | pH_arg | Kon0022140.1 | Pho_max |
| Kon0178890.1 | pH_arg | Kon0022460.1 | Pho_max |
| Kon0178950.1 | pH_arg | Kon0022470.1 | Pho_max |
| Kon0217210.1 | pH_arg | Kon0022720.1 | Pho_max |
| Kon0217540.1 | pH_arg | Kon0217970.1 | Pho_max |
| Kon0217570.1 | pH_arg | Kon0217980.1 | Pho_max |
| Kon0217580.1 | pH_arg | Kon0218580.1 | Pho_max |
| Kon0217590.1 | pH_arg | Kon0219480.1 | Pho_max |
| Kon0217650.1 | pH_arg | Kon0219910.1 | Pho_max |
| Kon0217710.1 | pH_arg | Kon0220040.1 | Pho_max |
| Kon0218000.1 | pH_arg | Kon0220110.1 | Pho_max |
| Kon0218010.1 | pH_arg | Kon0220120.1 | Pho_max |
| Kon0218290.1 | pH_arg | Kon0220400.1 | Pho_max |

Supplementary Table S6. GO enrichment results for pH_arg association genes

| ID | GO Term | Class | P-value | geneID | Count |
| --- | --- | --- | --- | --- | --- |
| GO:0071316 | cellular response to nicotine | Biological process | 0.00034 | Kon0219440.1/Kon0219760.1 | 2 |
| GO:0010603 | regulation of cytoplasmic mRNA processing body assembly | Biological process | 0.0004 | Kon0177580.1/Kon0217210.1 | 2 |
| GO:2001244 | positive regulation of intrinsic apoptotic signaling pathway | Biological process | 0.00047 | Kon0176220.1/Kon0217650.1/Kon0219440.1 | 3 |
| GO:0010826 | negative regulation of centrosome duplication | Biological process | 0.00075 | Kon0217210.1/Kon0217650.1 | 2 |
| GO:0046606 | negative regulation of centrosome cycle | Biological process | 0.00083 | Kon0217210.1/Kon0217650.1 | 2 |
| GO:0005154 | epidermal growth factor receptor binding | Molecular function | 0.0034 | Kon0176220.1/Kon0217210.1 | 2 |
| GO:0050700 | CARD domain binding | Molecular function | 0.01965 | Kon0220220.1 | 1 |
| GO:0001135 | obsolete RNA polymerase II transcription regulator recruiting activity | Molecular function | 0.0216 | Kon0220240.1 | 1 |
| GO:0008301 | DNA binding, bending | Molecular function | 0.02354 | Kon0217580.1 | 1 |
| GO:0016868 | intramolecular transferase activity, phosphotransferases | Molecular function | 0.02354 | Kon0217590.1 | 1 |
| GO:0001016 | RNA polymerase III transcription regulatory region sequence-specific DNA binding | Molecular function | 0.02741 | Kon0220240.1 | 1 |
| GO:0035375 | zymogen binding | Molecular function | 0.02741 | Kon0219760.1 | 1 |
| GO:0001134 | obsolete transcription regulator recruiting activity | Molecular function | 0.02934 | Kon0220240.1 | 1 |
| GO:0030346 | protein phosphatase 2B binding | Molecular function | 0.02934 | Kon0219440.1 | 1 |
| GO:0008097 | 5S rRNA binding | Molecular function | 0.03127 | Kon0177730.1 | 1 |
| GO:0043422 | protein kinase B binding | Molecular function | 0.03511 | Kon0219440.1 | 1 |
| GO:0055106 | ubiquitin-protein transferase regulator activity | Molecular function | 0.03702 | Kon0217650.1 | 1 |
| GO:0070851 | growth factor receptor binding | Molecular function | 0.03928 | Kon0176220.1/Kon0217210.1 | 2 |
| GO:1990381 | ubiquitin-specific protease binding | Molecular function | 0.04275 | Kon0217650.1 | 1 |
| GO:0061134 | peptidase regulator activity | Molecular function | 0.04528 | Kon0219440.1/Kon0219760.1 | 2 |
| GO:0016866 | intramolecular transferase activity | Molecular function | 0.04655 | Kon0217590.1 | 1 |
| GO:0019200 | carbohydrate kinase activity | Molecular function | 0.04844 | Kon0218000.1 | 1 |

Supplementary Table S7. GO enrichment results for T_max association genes

| ID | GO Term | Class | P-value | geneID | Count |
| --- | --- | --- | --- | --- | --- |
| GO:0044420 | obsolete extracellular matrix component | Cellular component | 0.00068 | Kon0176420.1/Kon0177250.1/Kon0219830.1 | 3 |
| GO:0005581 | collagen trimer | Cellular component | 0.00097 | Kon0176420.1/Kon0177250.1 | 2 |
| GO:0005788 | endoplasmic reticulum lumen | Cellular component | 0.00343 | Kon0176420.1/Kon0177250.1/Kon0177570.1/Kon0219830.1 | 4 |
| GO:0062023 | collagen-containing extracellular matrix | Cellular component | 0.00605 | Kon0176420.1/Kon0177250.1/Kon0219830.1 | 3 |
| GO:0005914 | spot adherens junction | Cellular component | 0.02003 | Kon0217590.1 | 1 |
| GO:0002199 | zona pellucida receptor complex | Cellular component | 0.02399 | Kon0217970.1 | 1 |
| GO:0031089 | platelet dense granule lumen | Cellular component | 0.02597 | Kon0175480.1 | 1 |
| GO:0005791 | rough endoplasmic reticulum | Cellular component | 0.02904 | Kon0176230.1/Kon0219830.1 | 2 |
| GO:0005604 | basement membrane | Cellular component | 0.03407 | Kon0177250.1/Kon0219830.1 | 2 |
| GO:0033270 | paranode region of axon | Cellular component | 0.03578 | Kon0178780.1 | 1 |
| GO:0044224 | juxtaparanode region of axon | Cellular component | 0.03578 | Kon0178780.1 | 1 |
| GO:0031012 | extracellular matrix | Cellular component | 0.04063 | Kon0176420.1/Kon0177250.1/Kon0219830.1 | 3 |
| GO:0042827 | platelet dense granule | Cellular component | 0.04162 | Kon0175480.1 | 1 |
| GO:0101031 | chaperone complex | Cellular component | 0.0455 | Kon0217970.1 | 1 |
| GO:0043083 | synaptic cleft | Cellular component | 0.04743 | Kon0219830.1 | 1 |
| GO:0016010 | dystrophin-associated glycoprotein complex | Cellular component | 0.04936 | Kon0217590.1 | 1 |
| GO:0090665 | glycoprotein complex | Cellular component | 0.04936 | Kon0217590.1 | 1 |
| GO:0050840 | extracellular matrix binding | Molecular function | 0.00865 | Kon0176420.1/Kon0219830.1 | 2 |
| GO:0043621 | protein self-association | Molecular function | 0.01569 | Kon0177570.1/Kon0219830.1 | 2 |
| GO:0005021 | vascular endothelial growth factor receptor activity | Molecular function | 0.01965 | Kon0177570.1 | 1 |
| GO:0033265 | choline binding | Molecular function | 0.01965 | Kon0219830.1 | 1 |
| GO:0016722 | oxidoreductase activity, acting on metal ions | Molecular function | 0.0216 | Kon0177300.1 | 1 |
| GO:0010181 | FMN binding | Molecular function | 0.02354 | Kon0177300.1 | 1 |
| GO:0016868 | intramolecular transferase activity, phosphotransferases | Molecular function | 0.02354 | Kon0217590.1 | 1 |
| GO:0035251 | UDP-glucosyltransferase activity | Molecular function | 0.02354 | Kon0176230.1 | 1 |
| GO:0045545 | syndecan binding | Molecular function | 0.02741 | Kon0177310.1 | 1 |
| GO:0070700 | BMP receptor binding | Molecular function | 0.02741 | Kon0178560.1 | 1 |
| GO:0008046 | axon guidance receptor activity | Molecular function | 0.02934 | Kon0177310.1 | 1 |
| GO:0070402 | NADPH binding | Molecular function | 0.02934 | Kon0177300.1 | 1 |
| GO:0042166 | acetylcholine binding | Molecular function | 0.03127 | Kon0219830.1 | 1 |
| GO:0030215 | semaphorin receptor binding | Molecular function | 0.03319 | Kon0177310.1 | 1 |
| GO:0038191 | neuropilin binding | Molecular function | 0.03319 | Kon0177310.1 | 1 |
| GO:0005044 | scavenger receptor activity | Molecular function | 0.03511 | Kon0175480.1 | 1 |
| GO:0046527 | glucosyltransferase activity | Molecular function | 0.03511 | Kon0176230.1 | 1 |
| GO:0005085 | guanyl-nucleotide exchange factor activity | Molecular function | 0.03791 | Kon0176360.1/Kon0177790.1/Kon0217560.1 | 3 |
| GO:0070696 | transmembrane receptor protein serine/threonine kinase binding | Molecular function | 0.04084 | Kon0178560.1 | 1 |
| GO:0071949 | FAD binding | Molecular function | 0.04084 | Kon0177300.1 | 1 |
| GO:0034237 | protein kinase A regulatory subunit binding | Molecular function | 0.04465 | Kon0177790.1 | 1 |
| GO:0016866 | intramolecular transferase activity | Molecular function | 0.04655 | Kon0217590.1 | 1 |

Supplementary Table S8. GO enrichment results of genes associated with multiple environmental variables

| ID | GO term | P-value | geneID |
| --- | --- | --- | --- |
| GO:0016331 | morphogenesis of embryonic epithelium | 0.00088 | *SCRIB/VAV3/EPB41L3* |
| GO:0090630 | activation of GTPase activity | 0.00132 | *SCRIB/VAV3* |
| GO:0035315 | hair cell differentiation | 0.00316 | *SCRIB/EPB41L3* |
| GO:0007043 | cell-cell junction assembly | 0.00326 | *SCRIB/EPB41L3* |
| GO:0007391 | dorsal closure | 0.00395 | *VAV3/EPB41L3* |
| GO:0090150 | establishment of protein localization to membrane | 0.00402 | *SCRIB/EPB41L3* |
| GO:0000290 | deadenylation-dependent decapping of nuclear-transcribed mRNA | 0.00455 | *PAN3* |
| GO:0019388 | galactose catabolic process | 0.00455 | *PGM5* |
| GO:0070202 | regulation of establishment of protein localization to chromosome | 0.00455 | *CCT7* |
| GO:0070203 | regulation of establishment of protein localization to telomere | 0.00455 | *CCT7* |
| GO:0098969 | neurotransmitter receptor transport to postsynaptic membrane | 0.00455 | *SCRIB* |
| GO:1902946 | protein localization to early endosome | 0.00455 | *SCRIB* |
| GO:1904851 | positive regulation of establishment of protein localization to telomere | 0.00455 | *CCT7* |
| GO:0072659 | protein localization to plasma membrane | 0.00485 | *SCRIB/EPB41L3* |
| GO:0098877 | neurotransmitter receptor transport to plasma membrane | 0.005 | *SCRIB* |
| GO:1903540 | establishment of protein localization to postsynaptic membrane | 0.005 | *SCRIB* |
| GO:1904816 | positive regulation of protein localization to chromosome, telomeric region | 0.00546 | *CCT7* |
| GO:0099632 | protein transport within plasma membrane | 0.00591 | *SCRIB* |
| GO:0099637 | neurotransmitter receptor transport | 0.00591 | *SCRIB* |
| GO:1904814 | regulation of protein localization to chromosome, telomeric region | 0.00591 | *CCT7* |
| GO:0007527 | adult somatic muscle development | 0.00636 | *EPB41L3* |
| GO:0010603 | regulation of cytoplasmic mRNA processing body assembly | 0.00636 | *PAN3* |
| GO:0038180 | nerve growth factor signaling pathway | 0.00636 | *KIDINS220* |
| GO:0099612 | protein localization to axon | 0.00636 | *EPB41L3* |
| GO:0001921 | positive regulation of receptor recycling | 0.00682 | *SCRIB* |
| GO:0043217 | myelin maintenance | 0.00682 | *EPB41L3* |
| GO:0097475 | motor neuron migration | 0.00727 | *SCRIB* |
| GO:0034329 | cell junction assembly | 0.00748 | *SCRIB/EPB41L3* |
| GO:1990778 | protein localization to cell periphery | 0.00843 | *SCRIB/EPB41L3* |
| GO:0099072 | regulation of postsynaptic membrane neurotransmitter receptor levels | 0.00863 | *SCRIB* |
| GO:0001700 | embryonic development via the syncytial blastoderm | 0.00874 | *VAV3/EPB41L3* |
| GO:0009913 | epidermal cell differentiation | 0.00889 | *SCRIB/EPB41L3* |
| GO:0006605 | protein targeting | 0.00895 | *EPB41L3/PAN3* |
| GO:0022612 | gland morphogenesis | 0.00895 | *SCRIB/EPB41L3* |
| GO:0071896 | protein localization to adherens junction | 0.00908 | *SCRIB* |
| GO:0006012 | galactose metabolic process | 0.00953 | *PGM5* |
| GO:0036010 | protein localization to endosome | 0.00953 | *SCRIB* |
| GO:0060088 | auditory receptor cell stereocilium organization | 0.00953 | *SCRIB* |
| GO:0060561 | apoptotic process involved in morphogenesis | 0.00999 | *SCRIB* |
| GO:0008362 | chitin-based embryonic cuticle biosynthetic process | 0.01044 | *EPB41L3* |
| GO:0021548 | pons development | 0.01044 | *SCRIB* |
| GO:0002093 | auditory receptor cell morphogenesis | 0.01089 | *SCRIB* |
| GO:0005978 | glycogen biosynthetic process | 0.01089 | *PGM5* |
| GO:0009250 | glucan biosynthetic process | 0.01089 | *PGM5* |
| GO:0032594 | protein transport within lipid bilayer | 0.01089 | *SCRIB* |
| GO:0000289 | nuclear-transcribed mRNA poly(A) tail shortening | 0.01224 | *PAN3* |
| GO:0016080 | synaptic vesicle targeting | 0.01269 | *SCRIB* |
| GO:0032288 | myelin assembly | 0.01269 | *EPB41L3* |
| GO:0045216 | cell-cell junction organization | 0.01278 | *SCRIB/EPB41L3* |
| GO:0007424 | open tracheal system development | 0.01296 | *VAV3/EPB41L3* |
| GO:0033564 | anterior/posterior axon guidance | 0.01314 | *SCRIB* |
| GO:0045199 | maintenance of epithelial cell apical/basal polarity | 0.01359 | *SCRIB* |
| GO:0050853 | B cell receptor signaling pathway | 0.01405 | *VAV3* |
| GO:0001919 | regulation of receptor recycling | 0.0145 | *SCRIB* |
| GO:0007339 | binding of sperm to zona pellucida | 0.0145 | *CCT7* |
| GO:0046037 | GMP metabolic process | 0.0145 | *SCRIB* |
| GO:0046710 | GDP metabolic process | 0.0145 | *SCRIB* |
| GO:1902414 | protein localization to cell junction | 0.0145 | *SCRIB* |
| GO:0042063 | gliogenesis | 0.01469 | *SCRIB/EPB41L3* |
| GO:0060857 | establishment of glial blood-brain barrier | 0.01495 | *EPB41L3* |
| GO:0071679 | commissural neuron axon guidance | 0.01495 | *SCRIB* |
| GO:0007016 | obsolete cytoskeletal anchoring at plasma membrane | 0.0154 | *EPB41L3* |
| GO:1903539 | protein localization to postsynaptic membrane | 0.0154 | *SCRIB* |
| GO:0034330 | cell junction organization | 0.01617 | *SCRIB/EPB41L3* |
| GO:0032212 | positive regulation of telomere maintenance via telomerase | 0.01629 | *CCT7* |
| GO:0035090 | maintenance of apical/basal cell polarity | 0.01629 | *SCRIB* |
| GO:0035036 | sperm-egg recognition | 0.01674 | *CCT7* |
| GO:1904358 | positive regulation of telomere maintenance via telomere lengthening | 0.01674 | *CCT7* |
| GO:0030890 | positive regulation of B cell proliferation | 0.01719 | *VAV3* |
| GO:0043547 | positive regulation of GTPase activity | 0.01772 | *SCRIB/VAV3* |
| GO:0006458 | 'de novo' protein folding | 0.01809 | *CCT7* |
| GO:0061343 | cell adhesion involved in heart morphogenesis | 0.01809 | *EPB41L3* |
| GO:0097120 | receptor localization to synapse | 0.01854 | *SCRIB* |
| GO:0038179 | neurotrophin signaling pathway | 0.01899 | *KIDINS220* |
| GO:0046940 | nucleoside monophosphate phosphorylation | 0.01944 | *SCRIB* |
| GO:0048857 | neural nucleus development | 0.01944 | *SCRIB* |
| GO:0060856 | establishment of blood-brain barrier | 0.01944 | *EPB41L3* |
| GO:0008544 | epidermis development | 0.01947 | *SCRIB/EPB41L3* |
| GO:0060117 | auditory receptor cell development | 0.01988 | *SCRIB* |
| GO:0060122 | inner ear receptor cell stereocilium organization | 0.01988 | *SCRIB* |
| GO:0061951 | establishment of protein localization to plasma membrane | 0.02078 | *SCRIB* |
| GO:0043552 | positive regulation of phosphatidylinositol 3-kinase activity | 0.02123 | *VAV3* |
| GO:0060027 | convergent extension involved in gastrulation | 0.02123 | *SCRIB* |
| GO:1901998 | toxin transport | 0.02212 | *CCT7* |
| GO:0032206 | positive regulation of telomere maintenance | 0.02257 | *CCT7* |
| GO:0090218 | positive regulation of lipid kinase activity | 0.02302 | *VAV3* |
| GO:0035089 | establishment of apical/basal cell polarity | 0.02346 | *SCRIB* |
| GO:0072499 | photoreceptor cell axon guidance | 0.02346 | *VAV3* |
| GO:0032210 | regulation of telomere maintenance via telomerase | 0.02391 | *CCT7* |
| GO:0060603 | mammary gland duct morphogenesis | 0.02391 | *SCRIB* |
| GO:0030593 | neutrophil chemotaxis | 0.02436 | *VAV3* |
| GO:0030888 | regulation of B cell proliferation | 0.02436 | *VAV3* |
| GO:0030032 | lamellipodium assembly | 0.0248 | *VAV3* |
| GO:0019991 | septate junction assembly | 0.02614 | *EPB41L3* |
| GO:0061162 | establishment of monopolar cell polarity | 0.02614 | *SCRIB* |
| GO:0061339 | establishment or maintenance of monopolar cell polarity | 0.02659 | *SCRIB* |
| GO:1904356 | regulation of telomere maintenance via telomere lengthening | 0.02659 | *CCT7* |
| GO:0009988 | cell-cell recognition | 0.02703 | *CCT7* |
| GO:0033692 | cellular polysaccharide biosynthetic process | 0.02703 | *PGM5* |
| GO:1990266 | neutrophil migration | 0.02703 | *VAV3* |
| GO:0000271 | polysaccharide biosynthetic process | 0.02792 | *PGM5* |
| GO:0030011 | maintenance of cell polarity | 0.02792 | *SCRIB* |
| GO:0038095 | Fc-epsilon receptor signaling pathway | 0.02837 | *VAV3* |
| GO:0050918 | positive chemotaxis | 0.02881 | *SCRIB* |
| GO:0000288 | nuclear-transcribed mRNA catabolic process, deadenylation-dependent decay | 0.0297 | *PAN3* |
| GO:0097581 | lamellipodium organization | 0.0297 | *VAV3* |
| GO:1902742 | apoptotic process involved in development | 0.0297 | *SCRIB* |
| GO:0042491 | inner ear auditory receptor cell differentiation | 0.03014 | *SCRIB* |
| GO:0071621 | granulocyte chemotaxis | 0.03014 | *VAV3* |
| GO:0035011 | melanotic encapsulation of foreign target | 0.03059 | *VAV3* |
| GO:0043551 | regulation of phosphatidylinositol 3-kinase activity | 0.03059 | *VAV3* |
| GO:0060026 | convergent extension | 0.03059 | *SCRIB* |
| GO:0040003 | chitin-based cuticle development | 0.03192 | *EPB41L3* |
| GO:0048557 | embryonic digestive tract morphogenesis | 0.03192 | *SCRIB* |
| GO:1903727 | positive regulation of phospholipid metabolic process | 0.03192 | *VAV3* |
| GO:0005977 | glycogen metabolic process | 0.03236 | *PGM5* |
| GO:0000186 | obsolete activation of MAPKK activity | 0.03369 | *KIDINS220* |
| GO:0006073 | cellular glucan metabolic process | 0.03369 | *PGM5* |
| GO:0019320 | hexose catabolic process | 0.03369 | *PGM5* |
| GO:0044042 | glucan metabolic process | 0.03369 | *PGM5* |
| GO:0097530 | granulocyte migration | 0.03369 | *VAV3* |
| GO:0038096 | Fc-gamma receptor signaling pathway involved in phagocytosis | 0.03413 | *VAV3* |
| GO:0034637 | cellular carbohydrate biosynthetic process | 0.03457 | *PGM5* |
| GO:0035010 | encapsulation of foreign target | 0.03457 | *VAV3* |
| GO:0038094 | Fc-gamma receptor signaling pathway | 0.03457 | *VAV3* |
| GO:0050871 | positive regulation of B cell activation | 0.03457 | *VAV3* |
| GO:0060443 | mammary gland morphogenesis | 0.03457 | *SCRIB* |
| GO:0002431 | Fc receptor mediated stimulatory signaling pathway | 0.03502 | *VAV3* |
| GO:0043550 | regulation of lipid kinase activity | 0.03502 | *VAV3* |
| GO:0098876 | vesicle-mediated transport to the plasma membrane | 0.03502 | *SCRIB* |
| GO:2000573 | positive regulation of DNA biosynthetic process | 0.03502 | *CCT7* |
| GO:0002433 | immune response-regulating cell surface receptor signaling pathway involved in phagocytosis | 0.03546 | *VAV3* |
| GO:0060119 | inner ear receptor cell development | 0.03634 | *SCRIB* |
| GO:0007229 | integrin-mediated signaling pathway | 0.03678 | *VAV3* |
| GO:0035006 | melanization defense response | 0.03678 | *VAV3* |
| GO:0061077 | chaperone-mediated protein folding | 0.03678 | *CCT7* |
| GO:0032204 | regulation of telomere maintenance | 0.03722 | *CCT7* |
| GO:0046365 | monosaccharide catabolic process | 0.03767 | *PGM5* |
| GO:0061180 | mammary gland epithelium development | 0.03767 | *SCRIB* |
| GO:0048010 | vascular endothelial growth factor receptor signaling pathway | 0.03899 | *VAV3* |
| GO:0035418 | protein localization to synapse | 0.03943 | *SCRIB* |
| GO:0006112 | energy reserve metabolic process | 0.03987 | *PGM5* |
| GO:0043113 | receptor clustering | 0.03987 | *SCRIB* |
| GO:1901068 | guanosine-containing compound metabolic process | 0.04075 | *SCRIB* |
| GO:0048566 | embryonic digestive tract development | 0.04207 | *SCRIB* |
| GO:1990090 | cellular response to nerve growth factor stimulus | 0.04295 | *KIDINS220* |
| GO:0009135 | purine nucleoside diphosphate metabolic process | 0.04382 | *SCRIB* |
| GO:0009179 | purine ribonucleoside diphosphate metabolic process | 0.04382 | *SCRIB* |
| GO:0097529 | myeloid leukocyte migration | 0.04382 | *VAV3* |
| GO:1903391 | regulation of adherens junction organization | 0.04426 | *VAV3* |
| GO:0000132 | establishment of mitotic spindle orientation | 0.04514 | *SCRIB* |
| GO:0006582 | melanin metabolic process | 0.04514 | *VAV3* |
| GO:0009185 | ribonucleoside diphosphate metabolic process | 0.04558 | *SCRIB* |
| GO:0009954 | proximal/distal pattern formation | 0.04558 | *EPB41L3* |
| GO:1990089 | response to nerve growth factor | 0.04558 | *KIDINS220* |
| GO:0060113 | inner ear receptor cell differentiation | 0.04602 | *SCRIB* |
| GO:0003382 | epithelial cell morphogenesis | 0.04733 | *SCRIB* |
| GO:0060563 | neuroepithelial cell differentiation | 0.04776 | *SCRIB* |
| GO:0035151 | regulation of tube size, open tracheal system | 0.04864 | *EPB41L3* |
| GO:0042059 | negative regulation of epidermal growth factor receptor signaling pathway | 0.04951 | *VAV3* |
| GO:0044264 | cellular polysaccharide metabolic process | 0.04995 | *PGM5* |
| GO:0050864 | regulation of B cell activation | 0.04995 | *VAV3* |
